# Hydrogen‐Bonding Networks Enabled by Trace Water for Morphological Design in Ternary Organic Solar Cells

**DOI:** 10.1002/advs.202517146

**Published:** 2026-01-04

**Authors:** Yue Ren, Ming‐Yue Sui, Yun Geng, Rui‐Cheng Qin, Ming‐Yang Li, Guang‐Yan Sun, Xin Xu

**Affiliations:** ^1^ Department of Chemistry Faculty of Science Institute of Quantum Science and Technology Yanbian University Yanji Jilin 133002 P. R. China; ^2^ Institute of Functional Material Chemistry, Faculty of Chemistry Northeast Normal University Changchun Jilin 130024 P. R. China; ^3^ Department of Collaborative Innovation Center of Chemistry for Energy Materials Shanghai Key Laboratory of Molecular Catalysis and Innovative Materials MOE Key Laboratory of Computational Physical Sciences Fudan University Shanghai 200438 P. R. China

**Keywords:** co‐solvent strategies, hydrogen‐bonding networks, molecular dynamics simulations, morphology designs, ternary organic solar cells

## Abstract

Morphology evolution is critical to the performance of functional materials, but strategies for its control remain largely empirical. Here, we identify a counterintuitive role of water (H_2_O) as a morphology‐regulating agent in ternary organic solar cells (OSCs), traditionally considered an impurity. Molecular dynamics simulations reveal that the dual hydrogen‐bonding capacity of H_2_O drives the formation of dynamic hydrogen‐bonding networks (HBNs). Continuous HBNs facilitate the migration of the third component into donor‐enriched domains through encapsulation, thereby stabilizing alloy‐like morphologies. While this HBN‐driven transition fails in single‐donor solvent systems such as ethanol, it extends to both fullerene and non‐fullerene blends in multi‐donor or acceptor environments. To render the mechanism applicable in organic processing, we adopted a co‐solvent strategy and identified a critical regime at a water‐to‐chloroform volume ratio of 0.06:1. At this threshold, trace H_2_O reproduces the alloy‐like behavior in neat H_2_O without compromising solubility, providing practical utility for device processing. Analyses of H_2_O containing CB, DMSO, and THF co‐solvents further confirm the general applicability of the HBN mechanism across distinct solvent environments. This work redefines H_2_O as a functional additive, establishes HBN engineering as a general framework for morphology control, and suggests broader implications for functional materials governed by weak interactions.

## Graphical abstract

Trace water transforms from an impurity to a design handle for ternary organic solar cells. During solvent evaporation, minimal amounts organize transient hydrogen‐bonding networks that steer component migration and set the morphology. The mechanism enables reproducible morphology locking and identifies a practical co‐solvent threshold near H_2_O:CF = 0.06:1 with relevance to scalable solution processing.

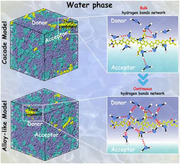

**Abbreviations**
AIM: atoms in molecules
BDT: benzo[1,2‐b:4,5‐b′]dithiophene
BTR: benzodithio‐phene terthiophene rhodamine
CB: chlorobenzene
CF: chloroform
COM: center‐of‐mass
DMSO: dimethyl sulfoxide
DR3TBDTT: 4,8 bis(5 alkylrhodanine 3 yl)benzo[1,2 b:4,5 b']dithiophene with three thienyl linkers
DR3TBDTT‐E: 4,8 bis(5 ethylrhodanine 3 yl)benzo[1,2 b:4,5 b']dithiophene with three thienyl linkers
DT: DR3TBDTT
DTE: DR3TBDTT‐E
EtOH: ethanol
GAFF: general amber force field
H_2_O: water
HBNs: hydrogen‐bonding networks
L8‐BO: 2,2'‐((2Z,2'Z)‐((12,13‐bis(2‐ethylhexyl)‐3,9‐(2‐butyloctyl)‐12,13‐dihydro‐[1,2,5]thiadiazolo[3,4‐e]thieno[2",3’':4’,5']thieno[2',3':4,5]pyrrolo[3,2‐g]thieno[2',3':4,5]thieno[3,2‐b]indole‐2,10‐diyl)bis(methanylylidene))bis(5,6‐difluoro‐3‐oxo‐2,3‐dihydro‐1H‐indene‐2,1‐diylidene))dimalononitrile
LA1: 4,8‐bis(5‐bromo‐2‐((3‐hexylthiophen‐2‐yl)methoxy)benzothiadiazole)‐2,6‐bis(4,7‐di(5‐hexylthiadiazole)‐2,1,3‐benzothiadiazole)
MD: molecular dynamics
MSD: mean square displacement
NPT: isobaric‐isothermal ensemble
OSCs: organic solar cells
PC_71_BM: 6,6‐phenyl‐C71‐butyric acid methyl ester
PCE: power conversion efficiency
PM6: also known as PBDB‐T‐2F, poly[(2,6‐(4,8‐bis(5‐(2‐ethylhexyl)thiophen‐2‐yl)benzo[1,2‐b:4,5‐b′]dithiophene))‐alt‐(5,5‐(1′,3′‐di‐2‐thienyl‐5′,7′‐bis(2‐ethylhexyl)benzo[1′,2′‐c:4′,5′‐c′]dithiophene‐4,8‐dione))]
PME: particle‐mesh ewald
RDF: radial distribution function
*r*_DT‐DTE_
: center‐of‐mass (COM) distance of the first nearest peak of radial distribution function (RDF) between the DT and DTE
*r*_DTE‐PC71BM_
: center‐of‐mass (COM) distance of the first nearest peak of radial distribution function (RDF) between the DTE and PC_71_BM
*r*_DT‐PC71BM_
: center‐of‐mass (COM) distance of the first nearest peak of radial distribution function (RDF) between the DT and PC_71_BM
RESP: restrained electrostatic potential
SASA: solvent‐accessible surface area
THF: tetrahydrofuran
vdW: van der Waals
Y6: 2,2'‐((2Z,2'Z)‐((12,13‐bis(2‐ethylhexyl)‐3,9‐diundecyl‐12,13‐dihydro‐[1,2,5]thiadiazolo[3,4‐e]thieno[2",3’':4’,5']thieno[2',3':4,5]pyrrolo[3,2‐g]thieno[2',3':4,5]thieno[3,2‐b]indole‐2,10‐diyl)bis(methanylylidene))bis(5,6‐difluoro‐3‐oxo‐2,3‐dihydro‐1H‐indene‐2,1‐diylidene))dimalononitrile
Y‐SeNF: 2,2'‐((((2,2'‐((((4,4,9,9‐Tetrakis(4‐(2‐butyloctyl)phenyl)‐4,9‐dihydro‐s‐indaceno[1,2‐b:5,6‐b']dithiophene‐2,7‐diyl))bis(selenopheno[3,2‐b]thiophene‐6,2‐diyl))bis(5‐(2‐butyloctyl)‐1H‐pyrrole‐4,2‐diyl))bis(methanylylidene))bis(5,6‐difluoro‐3‐oxo‐2,3‐dihydro‐1H‐indene‐2,1‐diylidene))dimalononitrile
co‐solvent strategy: Guest solvent H_2_O is introduced into the host solvent CF to construct co‐solvent system for film‐printing process.
DTE‐centered clusters: “DTE‐centered clusters” refer to the local molecular assemblies extracted from the larger blended cluster, where each one is defined with a DTE molecule at its core, encompassing the surrounding molecules within a specific cut‐off distance.
DT‐PC_71_BM: Parameters describing the interactions or geometric relationships between the DT and PC_71_BM molecules are denoted using the hyphenated notation DT‐PC_71_BM. For instance, *r*_DT‐PC71BM_ refers to the center‐of‐mass distance between them.
H_2_O···DT hydrogen bonds: Hydrogen bonds between H_2_O and DT molecules, denoted as “H_2_O···DT hydrogen bonds”, were identified based on geometric criteria: a donor‐acceptor distance of less than 3.5 Å and a donor‐hydrogen‐acceptor angle greater than 120°.
H_2_O···DTE hydrogen bonds: Hydrogen bonds between H_2_O and DTE molecules, denoted as “H_2_O···DTE hydrogen bonds”, were identified based on geometric criteria: a donor‐acceptor distance of less than 3.5 Å and a donor‐hydrogen‐acceptor angle greater than 120°.
H_2_O···PC_71_BM hydrogen bonds.: Hydrogen bonds between H_2_O and PC_71_BM molecules, denoted as “H_2_O···PC_71_BM hydrogen bonds”, were identified based on geometric criteria: a donor‐acceptor distance of less than 3.5 Å and a donor‐hydrogen‐acceptor angle greater than 120°.

## 1 Introduction

Morphology fundamentally governs the performance of advanced functional materials [1, 2, 3], spanning from nanoscale packing to mesoscale organization and thereby controlling excited‐state dynamics [4], carrier transport [5], and mechanical stability [6] across diverse systems [7, 8, 9]. In particular, organic solar cells (OSCs) are highly sensitive [10], since the power conversion efficiency (PCE) and stability depend on the optimal balance between phase separation and molecular ordering within the active layer [11, 12, 13]. Although morphology optimization through substitution [14, 15, 16], solvent engineering [17], and multicomponent strategies [18, 19] have enabled PCEs above 20% [20, 21, 22], current morphology design is guided more by empirical rules than by first principles, which limits the transferability and reproducibility of these strategies. Therefore, the urgent demand for a broadly applicable strategy to achieve rational control of morphology evolution becomes evident.

Intermolecular interactions provide a physically grounded route toward predictive control [23, 24]. Among them, hydrogen bonding offers directional and reversible forces that regulate molecular stacking and phase separation during solution processing [25, 26, 27]. Previous studies have demonstrated that deliberately introduced hydrogen‐bonding motifs can improve morphology stability and mechanical robustness in OSC blends [21, 22], highlighting the potential of hydrogen bonding as a multifunctional regulator of efficiency and durability [21]. Nevertheless, most of these approaches rely on specific molecular designs or dedicated additives, which limit their general applicability and complicate material synthesis.

Here, we propose a counterintuitive strategy in which trace amounts of water (H_2_O), traditionally regarded as an unwanted impurity, are repurposed as functional additives to regulate morphology evolution through dynamic hydrogen‐bonding networks (HBNs). During solvent evaporation [28], H_2_O molecules interact with carbonyl and fluorine motifs to initiate HBN formation, which serves as a molecular‐level switch guiding the blend toward distinct morphological arrangements and enabling controllable morphology engineering. Our molecular dynamics (MD) simulations [29] establish the mechanistic basis of this process and align with experimental observations [21]. By redefining the role of H_2_O from an unwanted impurity to a morphology‐regulating agent, this work establishes HBN engineering as a broadly applicable framework for active‐layer design, with implications for supramolecular assemblies [30, 31], environmentally benign processing [32, 33], and functional materials where weak interactions dictate performance [34].

## 2 Results and Discussion

### 2.1 Solvent‐Directed Control of the Guest Distribution

**FIGURE 1:**
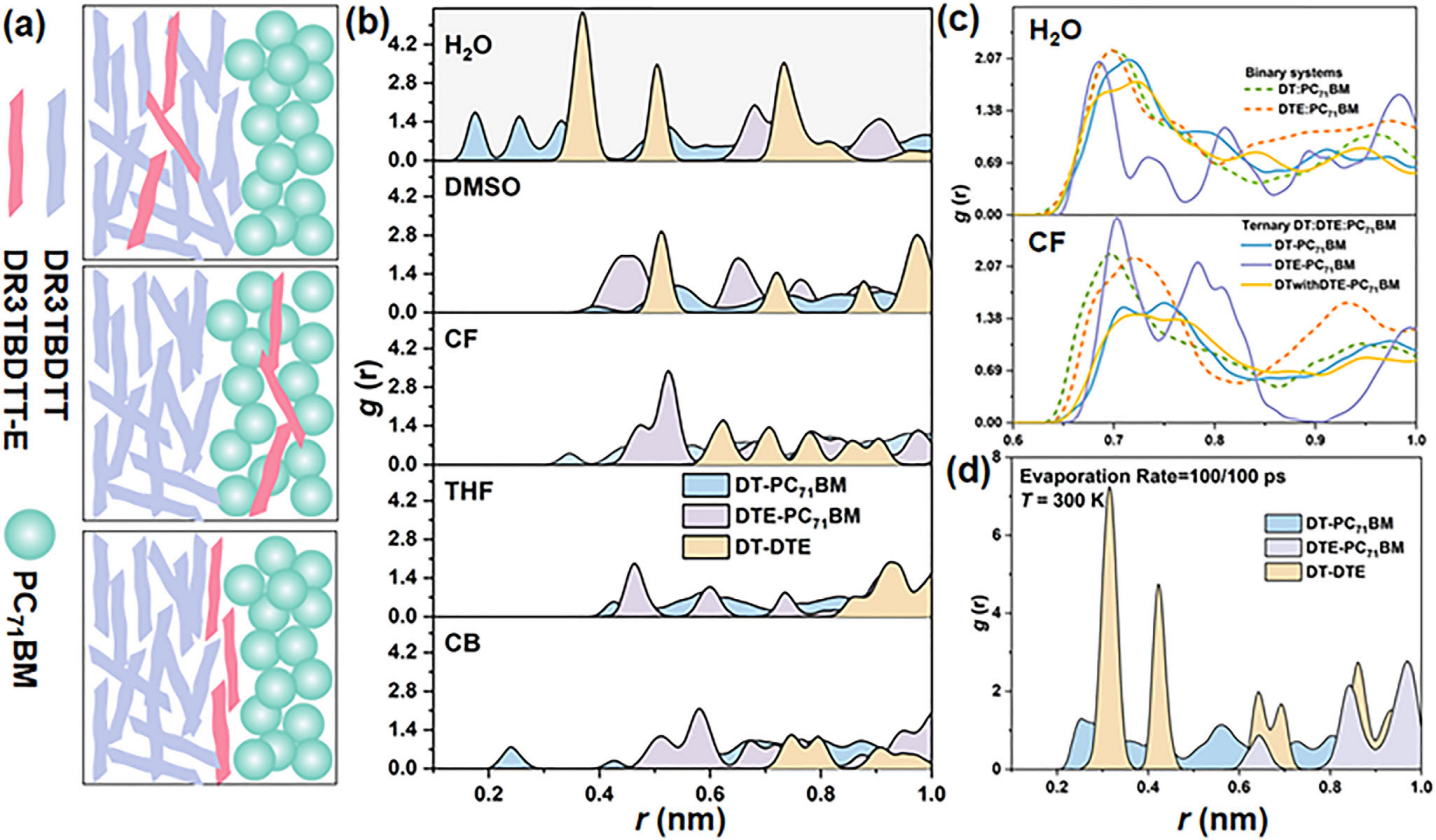
(a) Schematic illustration of DR3TBDTT‐E (DTE) spatial distributions in the ternary DT:DTE:PC_71_BM blends, including locations within the DR3TBDTT (DT) domains, within PC_71_BM domains, and at the DT and PC_71_BM interface. (b) COM RDFs of DT‐PC_71_BM, DTE‐PC_71_BM, and DT‐DTE in different solvents, DT‐PC_71_BM, DTE‐PC_71_BM represent the centroid distances between the PC_71_BM C_70_ unit and the BDT units originating from DT and from DTE, respectively. (c) COM RDFs of binary systems (DT:PC_71_BM and DTE:PC_71_BM) and ternary DT:DTE:PC_71_BM, DT withDTE‐PC_71_BM represent the center of mass distances between C_70_ and the BDT units originating from the DT‐DTE mixed environment. (d) RDFs of the ternary system with H_2_O evaporation (100 molecules/100 ps). Computational details and fragment definitions are provided in Sections S1 and S2.

The solvent environment governs the distribution of the guest component during ternary film formation. We selected representative solvents along a polarity gradient and performed MD simulations on DR3TBDTT (DT):DR3TBDTT‐E (DTE):PC_71_BM, owing to its tunable cascade (Figure 1a, bottom) and alloy‐like morphologies (Figure 1a, top) [35, 36]. Solvent effects were examined through center‐of‐mass (COM) radial distribution function (RDF) analysis in systems processed with H_2_O, dimethyl sulfoxide (DMSO), chloroform (CF), tetrahydrofuran (THF), and chlorobenzene (CB). Further computational details are provided in Sections S1 and S2. As shown in Figure 1b, only the H_2_O‐processed system deviates from the typical ordering of the first nearest neighbor peak (*r*_DT‐PC71BM_ < *r*_DTE‐PC71BM_ < *r*_DT‐DTE_). In H_2_O, the order is inverted, with DTE showing stronger spatial correlation with DT than with PC_71_BM, indicative of an alloy‐like morphology, whereas other solvents favor cascade arrangements. The comparisons of binary and ternary blends (Figure 1c) confirm the deviation. The ternary RDFs of DT and DTE lie between their respective binaries in CF, while in H_2_O, they nearly overlap, indicating full embedding of DTE within the DT domain. Changing the H_2_O evaporation temperature from 300 to 280 K or 320 K reverses the RDF ranking (Figure S3), evidencing a temperature‐dependent shift in thermodynamic preference. In contrast, reducing the H_2_O evaporation rate at 300 K preserves the baseline ranking (Figure 1d), indicating that temperature shifts the thermodynamic preference of local packing within the explored range, whereas moderate changes in evaporation rate do not alter the distance ordering.

### 2.2 Time‐Resolved Morphological Evolution

**FIGURE 2:**
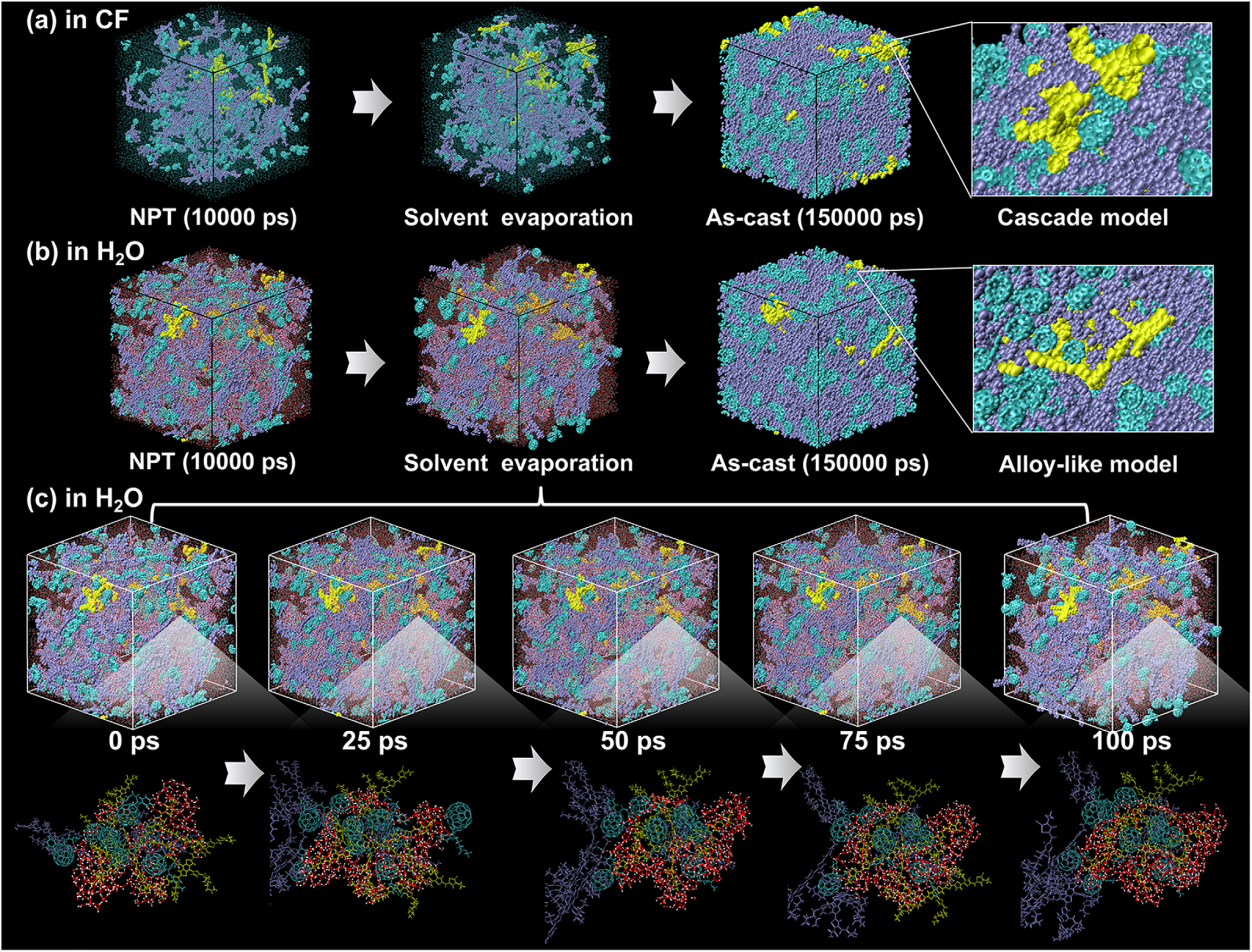
Morphological evolution of DT:DTE:PC_71_BM blends during the simulation process. Solvent evaporation produces a cascade‐type morphology in CF (a) and an alloy‐like model in H_2_O (b). (c) Time‐resolved snapshots during 100 ps in H_2_O reveal progressive solvent evaporation and hydrogen‐bonding network formation. Iceblue corresponds to DT, cyan to PC_71_BM, and yellow to DTE, while red dashed lines mark hydrogen bonds between H_2_O molecules.

Solvent choice dictates the morphological endpoint. To uncover the underlying dynamic transition process and the unique role of H_2_O, we further tracked the time‐resolved morphological evolution at the atomic level. Understanding the transformation from cascade to alloy‐like morphology requires direct insight into dynamic molecular rearrangement. The time‐resolved RDF analysis to track changes in the COM distance ordering for DT:DTE:PC_71_BM in H_2_O and CF solvents is shown in Figure 2. In CF, solvent evaporation produces a traditional morphology arrangement (Figure 2a), while in H_2_O, an alloy‐like morphology emerges (Figure 2b). RDF analyses capture the switch in distance ordering within the first 100 ps (Figure S6), consistent with the transformation observed in Figure 2a,b.

Beyond static RDF profiles, we aimed to comprehend the molecular basis of DTE migration and domain embedding. To quantify the migration of DTE into DT domains, mean square displacement (MSD) was calculated for individual DTE molecules over 0–100 ps (Figure S7). DTE‐centered clusters were defined using a cut‐off of 0.56 nm, derived from the first RDF peak relative to neighboring DT and PC_71_BM, and representative clusters in H_2_O were selected to trace directional motion and local contacts (Section S3.2). DTE contains ester‐functionalized BDT units that engage in hydrogen bonding with H_2_O, which biases its migration and embedding behavior compared with DT.

The time‐resolved snapshots in H_2_O (Figure 2c) delineate a temporal pathway of the transition. At 0 ps, the ternary blend exhibits a cascade model, where PC_71_BM partially disrupts the hydrogen bonding of H_2_O, resulting in fragmented and discontinuous HBNs. During 25–75 ps, redistribution of the components increases mixing and strengthens hydrogen bonding. The DTE clusters become increasingly encapsulated by interconnected O─H···O motifs, reflecting the development of continuous HBNs. At 100 ps, the cage‐like HBN consolidates and preferentially embeds DTE in DT‐rich regions, while separating it from PC_71_BM to establish alloy‐like uniformity. This evolution suggests that alloy‐like morphologies originate early and are stabilized not only by packing geometry but also by the maintenance of HBN continuity.

### 2.3 Mechanistic Origin: Formation of Continuous HBNs

**FIGURE 3:**
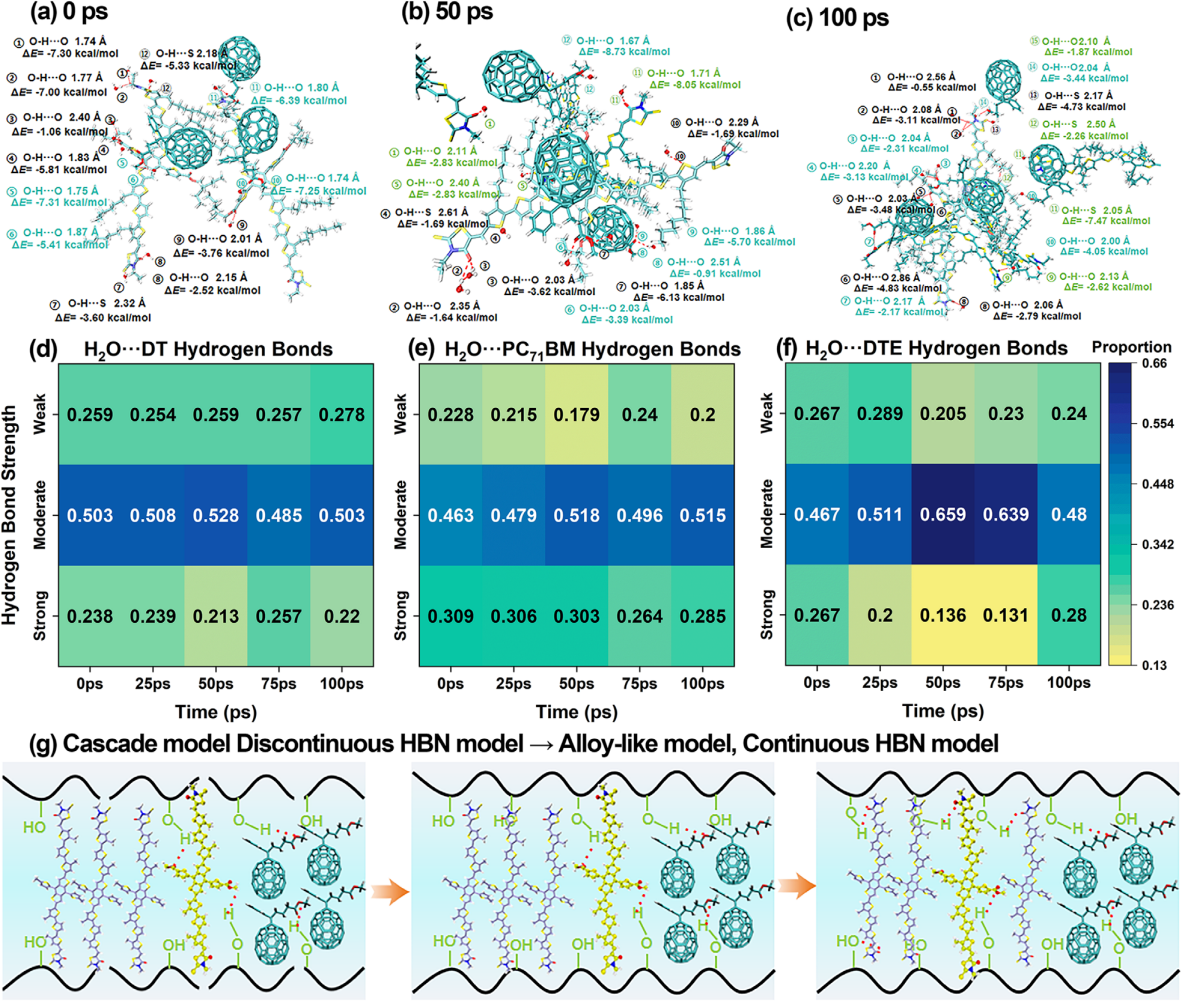
Hydrogen‐bond strength and network evolution during the solvent‐driven transition. (a‐c) Representative DTE‐centered clusters at 0, 50, and 100 ps with annotated O‐H···O distances and hydrogen‐bond energies to neighboring fragments. (d‐f) Heat maps showing the time evolution of hydrogen‐bond strength distributions for H_2_O···DT, H_2_O···PC_71_BM, and H_2_O···DTE hydrogen bonds; color denotes the normalized proportion within each strength range. (g) Schematic pathway from a fragmented to a continuous HBNs that stabilizes the alloy‐like morphology. Details of energy thresholds are given in Section S3.3.

To further investigate the microscopic mechanism for alloy‐like stabilization, we analyzed the local hydrogen‐bonding configurations and the evolution of bond strength within DTE‐containing regions. Energy partitioning based on Atoms in Molecules (AIM) analysis identifies strong, moderate, and weak hydrogen bonds, which are used to quantify the emergence of network continuity (Section S3.3). Representative clusters extracted from 0, 50, and 100 ps reveal the progression of local hydrogen‐bonding environments. At 0 ps in Figure 3a, the interactions between H_2_O and DTE are primarily weak to moderate due to disruption from PC_71_BM, forming fragmented and discontinuous HBNs. By 50 ps in Figure 3b, the number of strong hydrogen bonds decreases, indicating a transient destabilization of the network despite partial reorganization of H_2_O molecules. At 100 ps in Figure 3c, strong O‐H···O interactions reappear, and H_2_O molecules on the DTE surface form a cage‐like configuration, contributing to the reestablishment of a continuous HBN. The transient disruption reflects the highly dynamic yet resilient nature of HBNs. Additional weak and moderate bonds form between H_2_O and DT via carbonyl oxygen and sulfur atoms, driving DTE into the DT phase. Within this confined network, intermolecular hydrogen bonds among interior H_2_O molecules interact only weakly with external components. This behavior reflects the formation of a spatial boundary that structurally segregates DTE from PC_71_BM. This encapsulation stabilizes the alloy‐like morphology by physically isolating DTE within the DT phase.

Heat maps of H_2_O interacting with the three components (Figure 3d–f) show distinct behaviors. The distribution and continuity of strong hydrogen bonds vary across time and components. At 0 ps, 27% of H_2_O···DTE hydrogen bonds are already classified as strong, suggesting that the alloy‐like morphology may exist as a metastable configuration even before macroscopic blending occurs. However, these strong interactions between H_2_O and DTE undergo a transient decline in strength from 50 to 75 ps, indicating a temporary disruption of the HBN. By 100 ps, strong bonds reappear, particularly in interactions involving both DTE and DT, which reflects a dynamic reorganization process that stabilizes the continuous HBN and thereby preserves the alloy‐like morphology. In contrast, H_2_O···DT hydrogen bonds remain largely persistent over time, while H_2_O···PC_71_BM hydrogen bonds are consistently weak, underscoring their limited contribution to network formation. A conceptual representation of this transformation mechanism is presented in Figure 3g, where the cooperative effects of network rigidity and fluidity are illustrated as key contributors to morphological stabilization.

### 2.4 Dual versus Single Hydrogen Bonds

**FIGURE 4:**
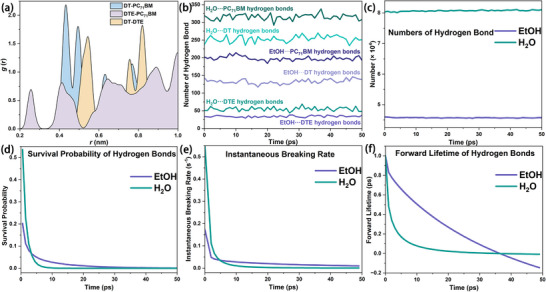
Comparative analysis of hydrogen‐bonding behavior between H_2_O and EtOH. (a) COM RDF maps in EtOH solvents. (b) Number of hydrogen bonds formed between H_2_O or EtOH and other blend components. (c) Total number of self‐hydrogen bonds among H_2_O or EtOH molecules. (d) Survival probability of hydrogen bonds over time. (e) Instantaneous breaking rate (ps^−1^) of hydrogen bonds, where H_2_O exhibits an earlier and sharper dissociation peak. (f) Forward lifetime (ps) of hydrogen bonds. H_2_O shows transient and regenerating hydrogen bonds, forming a dynamic HBN, while EtOH lacks such connectivity.

To distinguish the specific contribution of the dual hydrogen‐bonding ability of H_2_O from generic single‐donor solvent effects, ethanol (EtOH), containing only one hydrogen‐bond donor site, was used as a comparative solvent. As shown in Figure 4a and Section S4, EtOH fails to induce alloy‐like morphology, owing to the dispersed nature of its hydrogen‐bonding interactions. H_2_O forms many more hydrogen bonds with each component than ethanol (Figure 4b), and self‐hydrogen bonding in H_2_O far exceeds that in EtOH (Figure 4c), giving a denser network. This advantage is not only quantitative. Each H_2_O molecule provides two donating hydrogens and two lone pairs that act as hydrogen‐bonding acceptor sites, enabling multi‐directional and network spanning connectivity. But ethanol with a single hydroxyl mainly forms terminal contacts that limit propagation. As shown in Figure 4d–f, hydrogen bonds in H_2_O exhibit faster decay, sharper early‐time dissociation behavior, and shorter forward lifetimes compared to EtOH. These features indicate that individual hydrogen bonds in H_2_O are highly dynamic yet continuously reformed, yielding a flexible and reconfigurable HBN. Conversely, EtOH forms more static hydrogen bonds with limited mobility, failing to support network‐level rearrangement.

### 2.5 Generality Across Non‐Fullerene Ternary Blends

To assess generality, we examined three representative non‐fullerene ternary blends, PM6:L8‐BO:Y‐SeNF [37], PM6:Y6:LA1 [38], and PM6:BTR:Y6 [39] (Figures S1b–d and S4), which could encompass a range of chemical structures and molecular interactions, enabling a broad validation of the proposed HBN mechanism. COM RDFs in all cases (Figure S9) show the alloy‐like distance order in H_2_O, which suggests that the formation of alloy‐like morphology is not an isolated phenomenon. HBNs (Figure S10) can emerge in diverse donor‐acceptor frameworks under suitable solvent conditions. Specifically, time‐resolved analysis of PM6:L8‐BO:Y‐SeNF reveals a transition from cascade to alloy‐like morphology (Figure S11a) with directed motion of Y‐SeNF from PM6‐rich regions toward L8‐BO (Figure S11b). Concurrently, the number of PM6 and L8‐BO molecules surrounding Y‐SeNF was counted at 0 and 100 ps (Figure S11c), showing an increasing presence of L8‐BO in the local environment. Comparable trajectories are observed for PM6:Y6:LA1 and PM6:BTR:Y6 (Figure S11d–i). These observations confirm that the HBN‐driven mechanism is not limited to fullerene systems but is general to modern non‐fullerene ternary blends, which support the generality of the HBN mechanism.

### 2.6 A Practical Co‐Solvent Strategy and Threshold

#### 2.6.1 A Threshold Window in H_2_O:CF Environment

**FIGURE 5:**
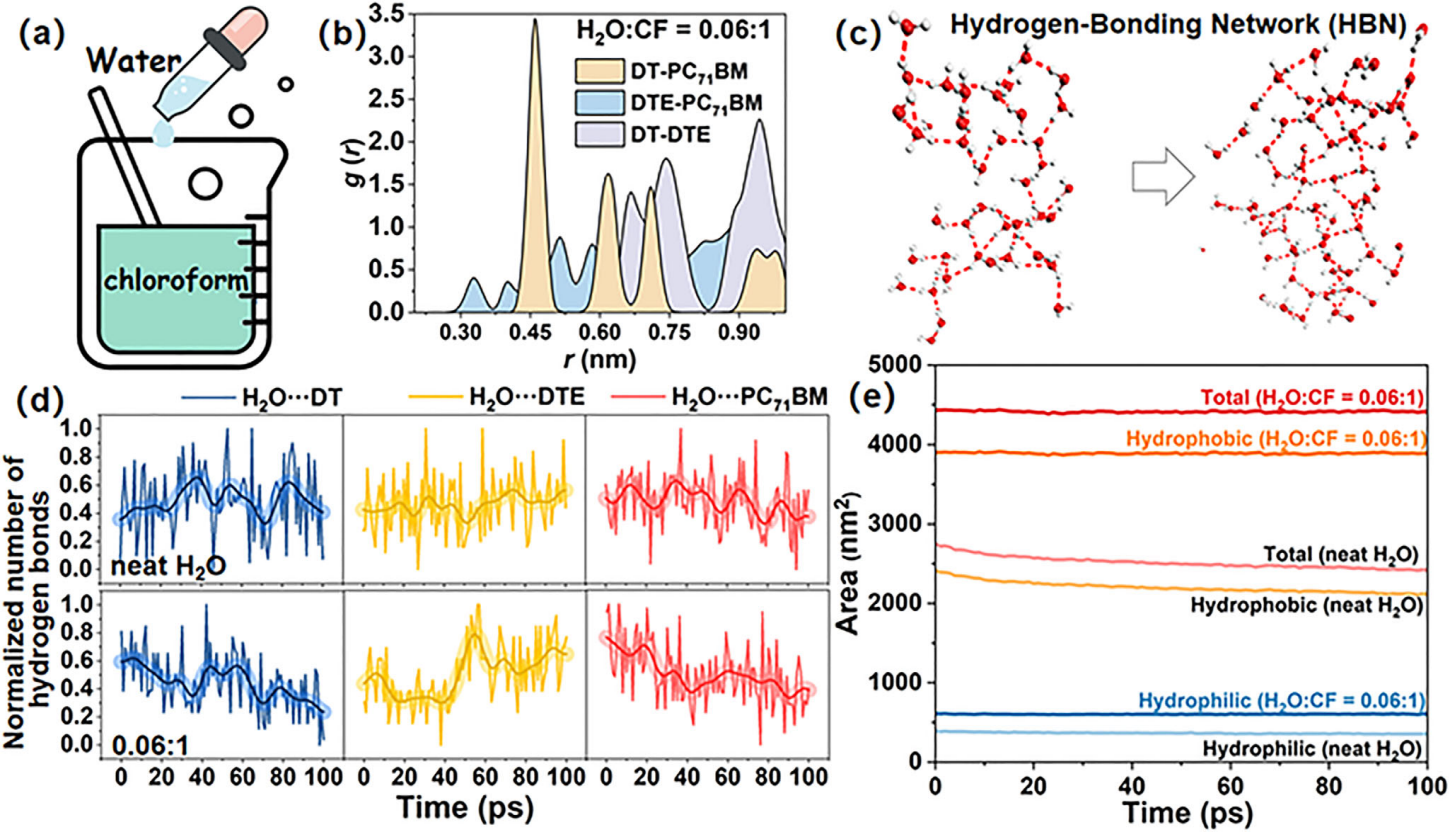
(a) Schematic illustration of the co‐solvent design strategy. (b) COM RDF progression as a function of H_2_O:CF = 0.06:1. (c) A HBN at the 0.06:1. (d) Temporal statistics of the total number of hydrogen bonds between H_2_O and each component under (top) neat H_2_O, and (bottom) H_2_O:CF = 0.06:1. (e) Solvent‐accessible surface area of a solute in the critical process from the cascade model to the alloy‐like model during MD simulations with neat and 0.06:1 solvent ratios. Total Solvent‐accessible surface area = hydrophobic surface area + hydrophilic surface area.

To translate the H_2_O‐driven mechanism into a practical processing route, we explored a co‐solvent condition (Figure 5a; Section 2.5) that preserves the hydrogen‐bonding benefit of H_2_O while maintaining solubility and volatility suitable for OSC fabrication. We mapped H_2_O:CF from 4:1, 1.50:1, 0.90:1, 0.67:1, 0.34:1, 0.25:1, and 0.15:1 to 0.06:1 (Figure 5b; Section S6.1). Reading from high to low H_2_O fraction, the COM RDF order follows a consistent progression. The ternary system at 4:1, 1.50:1, 0.90:1, and 0.67:1 passes through an alloy‐like window and then relaxes to a cascade outcome (Figure S14), while at 0.34:1, 0.25:1, and 0.15:1 the order remains CF‐like throughout, indicating a cascade morphology. At 0.06:1, the order coincides with that in H_2_O and remains alloy‐like at equilibrium (Figure 5c). This sequence indicates a gradual loss of hydrogen‐bonding continuity as the H_2_O fraction decreases, with a low fraction threshold near 0.06:1 where targeted HBNs are still sufficient to stabilize alloy‐like alignment. Similar to the H_2_O solvent, the continuous HBNs form in the 0.06:1 solvent ratio and drive DTE migration (Figure S15). It promotes the migration of DTE to the DT phase while reducing the contact with PC_71_BM. At 0.06:1, the continuous HBN forms persistent hydrogen bonds with DTE, resulting in limited migration over 0–100 ps (Figure S16). It indicates that HBN encapsulation hinders the migration of DTE. The bonding energy between the continuous HBNs and PC_71_BM is reduced, which leads PC_71_BM away from DTE.

Figure 5d compares hydrogen bond counts and shows that DTE with H_2_O and total network bonding are strongly enhanced relative to CF‐like conditions (Section S6.1.3). Figure 5e shows a higher solvent‐accessible surface area (SASA, Section S6.1.4) in CF with trace H_2_O than in neat H_2_O. This indicates that co‐solvents promote more extended and exposed conformations rather than compact ones. Hydrophobic segments remain compatible with CF, and polar sites reorient to recruit scarce H_2_O and form specific hydrogen bonds. Together, these observations support a practical co‐solvent window: a small H_2_O fraction near 0.06:1 seeds a transient, network‐spanning HBN early in drying, after which H_2_O is removed while the alloy‐like alignment persists. Such a co‐solvent window offers a realistic route to integrate H_2_O‐mediated morphology control into the OSC processing.

#### 2.6.2 Solvent‐Dependent Activation Windows of the Co‐Solvent Strategy

To clarify the effectiveness and robustness of the co‐solvent strategy, we performed a systematic scan of H_2_O fractions in another three representative H_2_O mixed solvent systems, namely H_2_O:CB, H_2_O:DMSO, and H_2_O:THF (Figures S17–S19), arranged in the order of solvent polarity shown in Figure 1. The cross‐solvent comparison indicates that HBNs induced alloy‐like models can be accessed across all examined systems, although the required compositions differ markedly (Section S6.2.2). These variations are consistent with the expected influences of H_2_O miscibility, intrinsic solvent polarity, and the competitive balance of hydrogen bonding between H_2_O and solvent.

In CF, the extremely low miscibility with H_2_O leads to localized HBNs that interact strongly with nearby donor and acceptor molecules, allowing trace H_2_O at 0.06:1 to stabilize an alloy‐like configuration. CB presents an intermediate activation regime, where only moderate H_2_O fractions offer sufficient association between H_2_O and solutes without inducing macroscopic segregation. In DMSO, strong solvent‐H_2_O hydrogen bonding keeps H_2_O highly dispersed at moderate or high H_2_O content, which suppresses the formation of interfacial H_2_O‐solute hydrogen bonds. Alloy‐like morphology appears only at very low H_2_O fractions such as 0.15:1 and 0.06:1, where isolated H_2_O molecules are sufficiently unsolvated to engage in targeted interfacial hydrogen bonding and initiate HBN formation. THF exhibits the opposite dependence. Since THF is highly miscible with H_2_O, hydrogen‐bonding motifs are dispersed at low H_2_O levels, and only H_2_O‐rich conditions restore the connectivity needed to activate the HBN mechanism (Section S6.2.3).

Overall, the HBN mechanism operates across a broad solvent spectrum, whereas the activation window is governed by solvent polarity, H_2_O miscibility, and the hierarchical competition among hydrogen‐bonding interactions. Such a solvent‐dependent activation landscape provides a practical design space in which trace H_2_O fractions act as effective morphological additives without compromising solution processability.

## 3 Conclusion

In conclusion, our study establishes H_2_O as a functional additive that transforms from an impurity into a key regulator of morphology in ternary OSCs. Rather than being dictated by solvent evaporation kinetics, morphology evolution is shown to be governed by the thermodynamic continuity of HBNs. This insight clarifies the distinct role of H_2_O relative to other protic solvents and highlights HBN engineering as a broadly applicable principle. Systematic mapping further reveals a threshold regime where even trace amounts of H_2_O can seed network continuity and lock in stable morphology, which offers a practical handle for rational processing strategies in ternary OSCs.

## 4 Methods

All the all‐atom MD simulations were performed using the GROMACS 2018 software package [40]. The atom types and intermolecular interaction parameters for all molecules were derived from the general amber force field (GAFF) [41] using the restrained electrostatic potential (RESP) fitting method. Under the NPT ensemble, a leap‐frog integrator [42] with a time step of 1 fs and 3D periodic boundary conditions was employed. A spherical cut‐off of 1.2 nm was used for the summation of van der Waals (vdW) interactions, and the particle‐mesh Ewald (PME) [43] solver was employed for long‐range Coulomb interactions. Molecular structures and detailed definitions are provided in the Sections S1 and S2. Hydrogen bond energies were obtained from quantum chemical calculations with Gaussian 16 [44] and analyzed with Multiwfn 3.8 (dev) [45, 46, 47] in Section S3.3, which underpins the continuity analysis in Section 2.3. For clarity, all abbreviations used in this work are listed with their full names or definitions in the Appendix of the Supporting Information.

## Conflicts of Interest

The authors declare no conflicts of interest.

## Supporting information




**Supporting File**: advs73561‐sup‐0001‐SuppMat.docx.

## Data Availability

The data that support the findings of this study are available in the supplementary material of this article.
