# Supplementary material for: Hydrogen‐Bonding Networks Enabled by Trace Water for Morphological Design in Ternary Organic Solar Cells
Source: Adv Sci (Weinh). 2026 Jan 4;13(14):e17146. doi: 10.1002/advs.202517146 (PMC12970264; doi:10.1002/advs.202517146)
Supplement: Supplementary file 1 — Supporting File: advs73561‐sup‐0001‐SuppMat.docx. [file ADVS-13-e17146-s001.docx]

Supporting Information

**Hydrogen-Bonding Networks Enabled by Trace Water for Morphological Design in Ternary Organic Solar Cells**

Yue Ren,^[a]^ Ming-Yue Sui,^[a]^ Yun Geng,^[b]^ Rui-Cheng Qin,^[a]^ Ming-Yang Li,^[a]^* Guang-Yan Sun,^[a]^* Xin Xu^[a, c]^*

[a] Yue Ren, Ming-Yue Sui, Rui-Cheng Qin, Ming-Yang Li, Guang-Yan Sun, Xin Xu

Department of Chemistry, Faculty of Science, Institute of Quantum Science and Technology, Yanbian University, Yanji 133002, Jilin, P. R. China
E-mail: myli6@ybu.edu.cn (Ming-Yang Li, orcid.org/0009-0009-6802-2197); gysun@ybu.edu.cn (Guang-Yan Sun, orcid.org/0000-0002-5236-9753);

xxchem@fudan.edu.cn (Xin Xu, orcid.org/0000-0002-5247-2937)

[b] Yun Geng

Institute of Functional Material Chemistry, Faculty of Chemistry, Northeast Normal University, Changchun 130024, Jilin, P. R. China

[c] Xin Xu

Department of Collaborative Innovation Center of Chemistry for Energy Materials, Shanghai Key Laboratory of Molecular Catalysis and Innovative Materials, MOE Key Laboratory of Computational Physical Sciences, Fudan University, Shanghai 200438, P. R. China

**Outline**

[Section S1 Models 3](#_Toc317)

[Section S1.1 Fullerene Blends as Study Models 3](#_Toc26208)

[Section S1.2 Non-Fullerene Blends as Validation Models 3](#_Toc20098)

[Section S2 Molecular Dynamics Simulation 4](#_Toc13870)

[Section S2.1 Initial Simulation Setup and Blend Compositions 4](#_Toc10979)

[Section S2.2 Simulation Workflow 4](#_Toc26056)

[Section S2.3 Solvent Effects 5](#_Toc21883)

[Section S2.3.1 Polarity Effect 5](#_Toc8935)

[Section S2.3.2 Binary Blend Comparison 6](#_Toc11269)

[Section S2.3.3 Control Tests: Temperature and Evaporation-Rate Effects in H](#_Toc10170)_[2](#_Toc10170)_[O 6](#_Toc10170)

[Section S2.4 Non-Fullerene Blends 7](#_Toc28132)

[Section S2.5 Blend Ratios and Evaporation Protocols 7](#_Toc13369)

[Section S3 Dynamic Alloy-like Morphological Evolution in H](#_Toc2223)_[2](#_Toc2223)_[O 9](#_Toc2223)

[Section S3.1 Identification of Critical Process 9](#_Toc32542)

[Section S3.2 Migration Distance and Stacking Molecules Statistics of DTE 9](#_Toc16844)

[Section S3.3 Hydrogen-Bonding Network 10](#_Toc17280)

[S3.3.1 Hydrogen Bond Strength Classification 10](#_Toc28184)

[S3.3.2 Statistical Thresholding and Classification 11](#_Toc30820)

[Section S4 Dynamic Alloy-like Morphological Evolution in EtOH 12](#_Toc12216)

[Section S5 Hydrogen-Bonding Network in Non-Fullerene Systems 13](#_Toc27075)

[Section S5.1 Visualization of Morphological Change Process 13](#_Toc13186)

[Section S5.2 Dynamic Elucidation of Morphological Change Process 13](#_Toc26323)

[Section S6 Co-solvent Strategy 17](#_Toc29346)

[Section S6.1 Co-solvent Strategy of H](#_Toc6046)_[2](#_Toc6046)_[O and CF 17](#_Toc6046)

[Section S6.1.1 Radial Distribution Functions for the Final Equilateral Ternary Blends of mixed H](#_Toc25186)_[2](#_Toc25186)_[O with CF 17](#_Toc25186)

[Section S6.1.2 Dynamic Elucidation of Morphological Change Process 18](#_Toc23582)

[Section S6.1.3 Hydrogen Bond Counts 19](#_Toc2881)

[Section S6.1.4 Solvent-Accessible Surface Area (SASA) 19](#_Toc27007)

[Section S6.2 The Extension of the Co-solvent Strategy 20](#_Toc2497)

[Section S6.2.1 Radial Distribution Functions for the Final Equilateral Ternary Blends of mixed H](#_Toc15929)_[2](#_Toc15929)_[O with CB, DMSO and THF 20](#_Toc15929)

[Section S6.2.2 Dynamic Elucidation of Morphological Change Process of mixed H](#_Toc20107)_[2](#_Toc20107)_[O with CB, DMSO and THF 23](#_Toc20107)

[Section S6.2.3 Unique Behavior of THF 26](#_Toc3094)

[Section S7 References 27](#_Toc24802)

[Appendix: List of Abbreviations and Their Full Names or Definitions 29](#_Toc28522)

# Section S1 Models

## Section S1.1 Fullerene Blends as Study Models

DR3TBDTT:DR3TBDTT-E:PC_71_BM^[1]^, as shown in **Figure S1a**, DR3TBDTT named DT is the host donor (D_1_), PC_71_BM is the acceptor, and DR3TBDTT-E named DTE with with ester groups is guest donor (D_2_) as the third component. The inclusion of PC_71_BM, a fullerene derivative with isotropic geometry and well-established phase behavior, enables a mechanistic study grounded in both extensive experimental data and theoretical simulation benchmarks. DT:DTE:PC_71_BM denotes the ternary blend configuration, with notations such as DT-DTE referring to the interfacial region between DT and DTE. Specifically, DT-DTE-PC_71_BM denotes spatial arrangements where DTE distribution is located between DT and PC_71_BM.

## Section S1.2 Non-Fullerene Blends as Validation Models

As shown in **Figure S1b-d**, PM6:L8-BO:Y-SeNF^[2]^ and PM6:Y6:LA1^[3]^ represent 1D:2A involving one polymer donor and two small-molecule acceptors. PM6:BTR:Y6^[4]^ comprises a polymer donor (PM6), a small-molecule donor (BTR), and a non-fullerene acceptor (Y6).


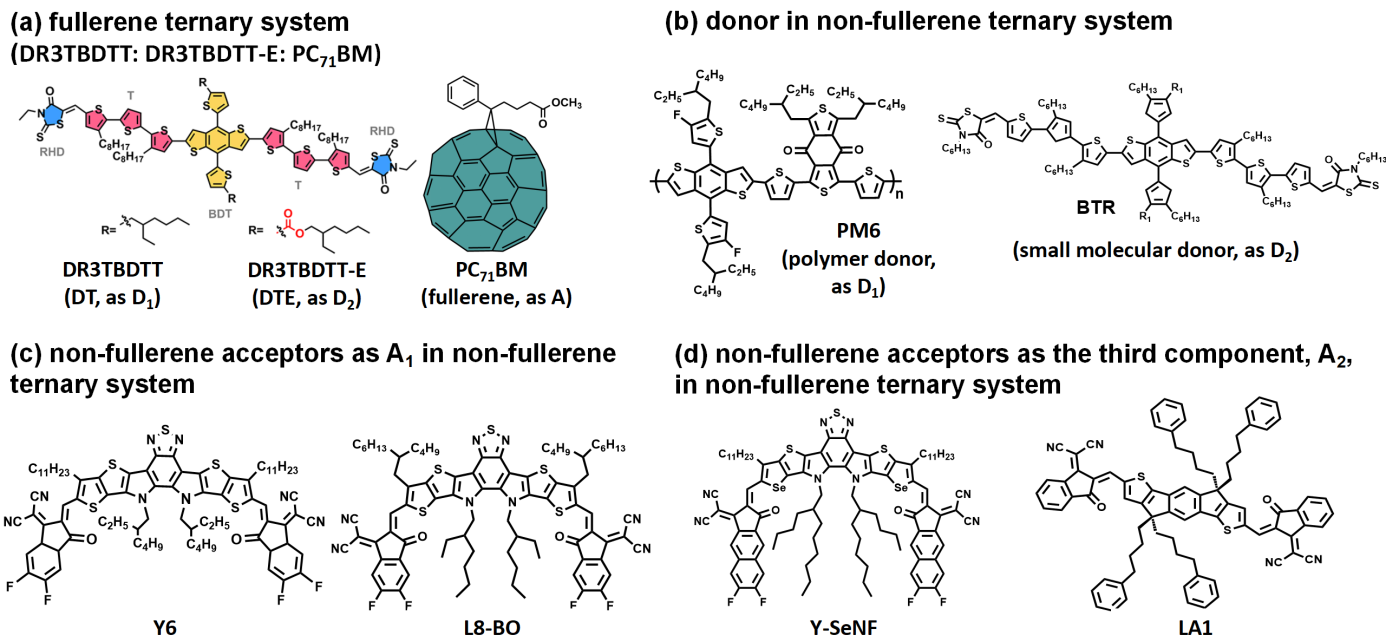


**Figure S1**. Chemical structures of study and validation models. (a) DR3TBDTT (DT), DR3TSBDTT-E (DTE), and PC_71_BM in fullerene system. (b) PM6 and BTR in non-fullerene systems. (c) Y6 and L8-BO in non-fullerene systems. (d) Y-SeNF and LA1 in non-fullerene systems.

# Section S2 Molecular Dynamics Simulation

## Section S2.1 Initial Simulation Setup and Blend Compositions

To maintain the experimental weight ratios (0.9:0.1:0.8), 124:13:200 molecules of DT:DTE:PC_71_BM were used for each blend in a solution containing 50,000 solvent molecules within a cubic box measuring 35 nm on each side. PM6:L8-BO:Y-SeNF includes 54 PM6, 178 L8B, and 30 Y-SeNF molecules; PM6:Y6:LA1 includes 37 PM6, 124 Y6, and 30 LA1 molecules, and PM6:BTR:Y6 includes 79 PM6, 336 L8B, and 10 Y-SeNF molecules. Chloroform (CF) and water (H_2_O) were two kinds of solvents. CF was chosen as a benchmark solvent owing to its well-established cascade-type morphology, confirmed in both experimental and theoretical studies.^[5-6]^ H_2_O as an external medium to promote intermolecular non-covalent interactions was introduced to probe solvent-induced morphological transitions.

## Section S2.2 Simulation Workflow


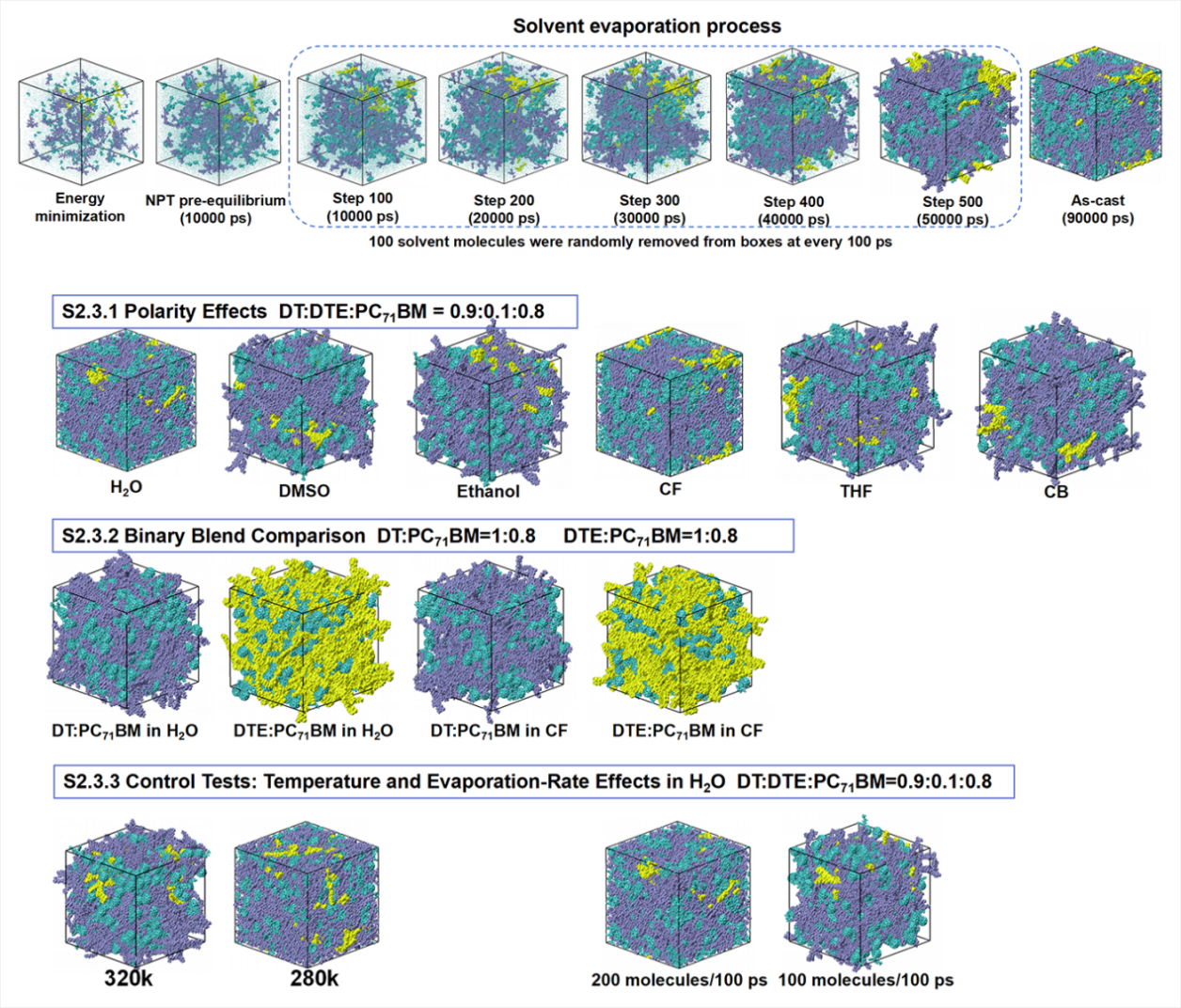


**Figure S2**. Workflow of simulating the blending morphologies of DT:DTE:PC_71_BM. Representative and final snapshots of the blending morphologies under different processing conditions and solvent. Icebule represents the host donor DT, cyan represents the acceptor PC_71_BM, and yellow represents the third component DTE (guest donor).

We simulated the solution processing of blend films using non-equilibrium molecular dynamics (MD) simulations. The atom types and intermolecular interaction parameters for all molecules were derived from the general amber force field (GAFF). The GAFF employs atomic charges and Lennard-Jones potentials to describe electrostatic and van der Waals interactions and has been validated for numerous organic molecules, including π-systems. The simulated process followed the steps in **Figure S2**: 1) conducting energy minimization and a 10 ns NPT equilibration to reduce the system energies at 300 K and 1 bar and bring molecules into close proximity. NPT ensemble were carried with the Berendsen pressure barostat and the Velocity Rescaling^[7]^ temperature thermostat. The Berendsen barostat and Velocity Rescaling thermostat were employed for this initial equilibration due to their efficient and stable coupling algorithm, which is well-suited for rapidly relaxing the system before the production phase; 2) employing a quasi-equilibrium approach^[8]^ to model the solvent evaporation process, in which 200 solvent molecules were randomly removed from the simulated boxes at every 100 ps to evaporate 50000 solvent molecules. This method was deliberately chosen over simpler alternatives. The quasi-equilibrium approach allows the system to relax between incremental removal events, better mimicking a physical drying process and enabling the development of more realistic, anisotropic morphologies. Therefore, solvent evaporation is modeled by deleting solvent molecules randomly from a bulk solution under 3D periodic boundary conditions; 3) equilibrating of the dried films for 90 ns, followed by the final 5 ns for data collection at 300 K and 1 bar with Parrinello-Rahman^[9]^ pressure barostat. The barostat was switched to more accurate Parrinello-Rahman for this production phase to correctly sample the NPT ensemble and capture the true pressure fluctuations, which is crucial for obtaining accurate thermodynamic and structural properties. Equilibration of the dried films for 90 ns was performed after a 10 ns NPT equilibration without solvent extraction. This work examines the fundamental factors influencing morphological dynamic during the thin film preparation process, including solvent addition and evaporation, solvent polarity, evaporation rate, and co-solvent effects. After as cast, the equilibrium blending morphology under different conditions is shown in **Figure S2**.

## Section S2.3 Solvent Effects

### Section S2.3.1 Polarity Effect

The selected solvents span a wide range of dielectric constants and boiling points, including H_2_O (*ε* = 10.2, 100 °C), DMSO (7.2, 189 °C), CF (4.1, 61 °C), THF (4.0, 66 °C), and chlorobenzene (2.7, 132 °C),^[10]^ which enable a systematic examination of solvation effects on morphology. As shown in **Figure 1b**, only the system processed with H_2_O deviates from the typical RDF ordering, in which *r*_DT-PC71BM_ < *r*_DTE-PC71BM_ < *r*_DT-DTE_. In the case of H_2_O, the order is different, with DTE showing a closer spatial correlation with DT than with PC_71_BM. This observation strongly suggests that H_2_O solvent may induce a specific morphology characteristic similar to the alloy-like model, while the others prefer to form cascade models.

### Section **S2.3.2 Binary Blend Comparison**

**Figure 1c:** The binary DT:PC_71_BM and DTE:PC_71_BM systems were simulated at a ratio of 1:0.8, both in CF and H_2_O solvent. In CF, the ternary RDFs of DT and DTE reside between their respective binaries, suggesting partial intermixing and interfacial distribution of DTE. In contrast, in H_2_O, COM *r* value of the ternary film is not located between the two binary films. Meanwhile, the RDF map contacts between the BDT moiety of DT and the C70 cage of PC_71_BM completely overlays the corresponding RDF representing the BDT moieties jointly contributed by DT and DTE toward the C70 cage, indicating that more DTE and DT tend to form an alloy-like model within a blend domain. There is only a small amount present outside the DT phase, which is different from the results in CF solvents. The DT and DTE RDFs in the ternary film almost overlap, indicating that DTE is fully embedded in the DT domain. This provides further evidence for an H_2_O-induced alloy-like configuration.

### Section **S2.3.3 Control Tests: Temperature and Evaporation-Rate Effects in H_2_O**

**Control Test I: Temperature Effect in H_2_O (Figure S3)**

At 300 K, the DT:DTE:PC_71_BM blend processed in H_2_O exhibits the RDF sequence *r*_DT-PC71BM_ < *r*_DT-DTE_ < *r*_DTE-PC71BM_. Holding the protocol unchanged while changing the temperature to 280 K and 320 K flips the sequence to *r*_DT-PC71BM_ < *r*_DTE-PC71BM_ < *r*_DT-DTE_. This inversion evidences a temperature-dependent thermodynamic shift in the relative stability of DT-DTE versus DTE-PC₇₁BM contacts.


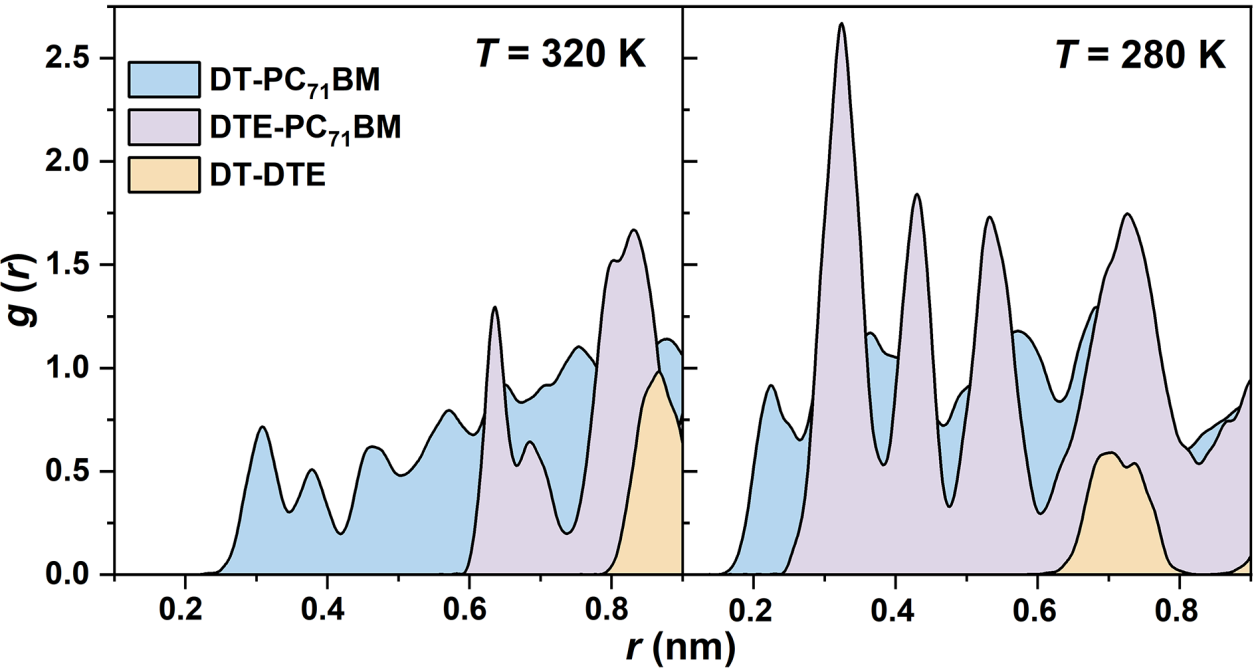


**Figure S3**. Center-of-mass (COM) radial distribution functions (RDFs) maps in H_2_O solvents for the DT-PC_71_BM (blue), DTE-PC_71_BM (purple), and DT-DTE (orange) of ternary systems at 320 K and 280 K of evaporation temperature.

**Control Test II: Evaporation-Rate Effect in H_2_O (Figure 1d)**

In contrast, reducing the evaporation rate at 300 K does not change the final ordering. To assess the potential influence of kinetic effects, solvent evaporation rates in H_2_O were varied from 200 molecules per 100 ps to 100 molecules per 100 ps. The RDF profiles obtained under both conditions were essentially identical, indicating that the H_2_O-induced morphology is not governed by kinetic evaporation rates but instead by intrinsic thermodynamic solvent-solute interactions. This confirms that the role of H_2_O is intrinsic rather than process-dependent.

## Section S2.4 Non-Fullerene Blends


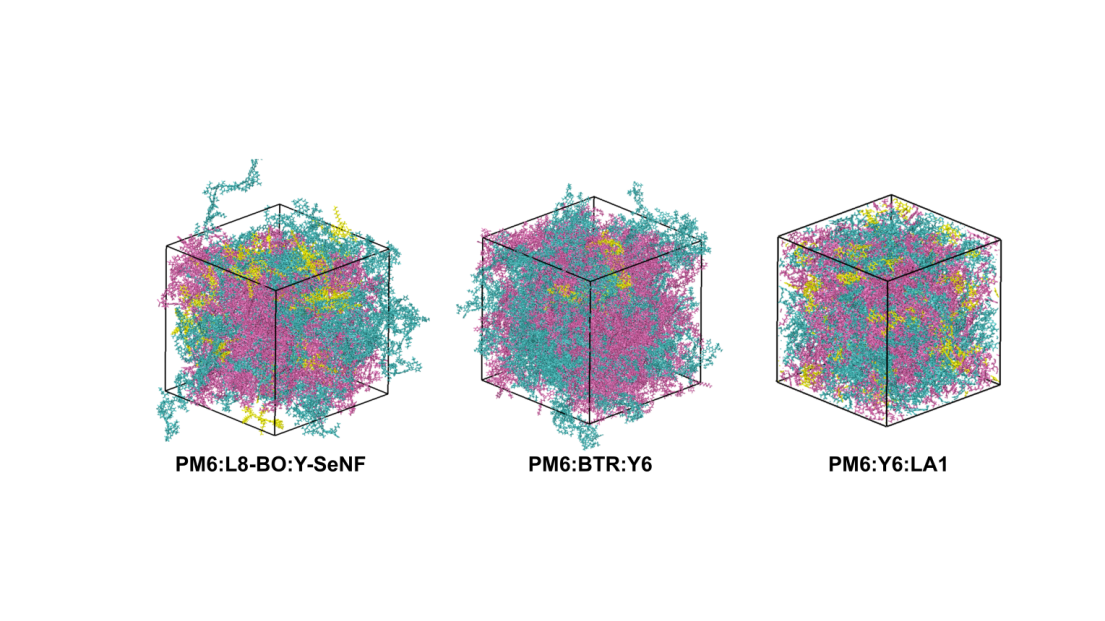


**Figure S4**. Representative and final snapshots of the blending morphologies for PM6:L8-BO:Y-SeNF, PM6:Y6:LA1, and PM6:BTR:Y6 in H_2_O solvent. Cyan represents the donor PM6, magenta represents the acceptors L8-BO and Y6, and yellow represents the third components Y-SeNF, LA1.

## Section S2.5 Blend Ratios and Evaporation Protocols

Eight ratios of 4:1, 1.50:1, 0.90:1, 0.67:1, 0.34:1, 0.25:1, 0.15:1 and 0.06:1 (v-v) were simulated based on 50000 solvent molecules to consider co-solvent strategy.

**Table S1**. Molecular numbers of H_2_O and solvents (including CF, CB, DMSO and THF) at different initial volume ratios (v/v) with a total of 50,000 molecules

| v-v | 4:1 | 1.50:1 | 0.90:1 | 0.67:1 | 0.34:1 | 0.25:1 | 0.15:1 | 0.06:1 |
| --- | --- | --- | --- | --- | --- | --- | --- | --- |
| H_2_O | 47333 | 43469 | 40000 | 37368 | 30000 | 26295 | 20000 | 10000 |
| Solvent | 2667 | 6531 | 10000 | 12632 | 20000 | 23705 | 30000 | 40000 |


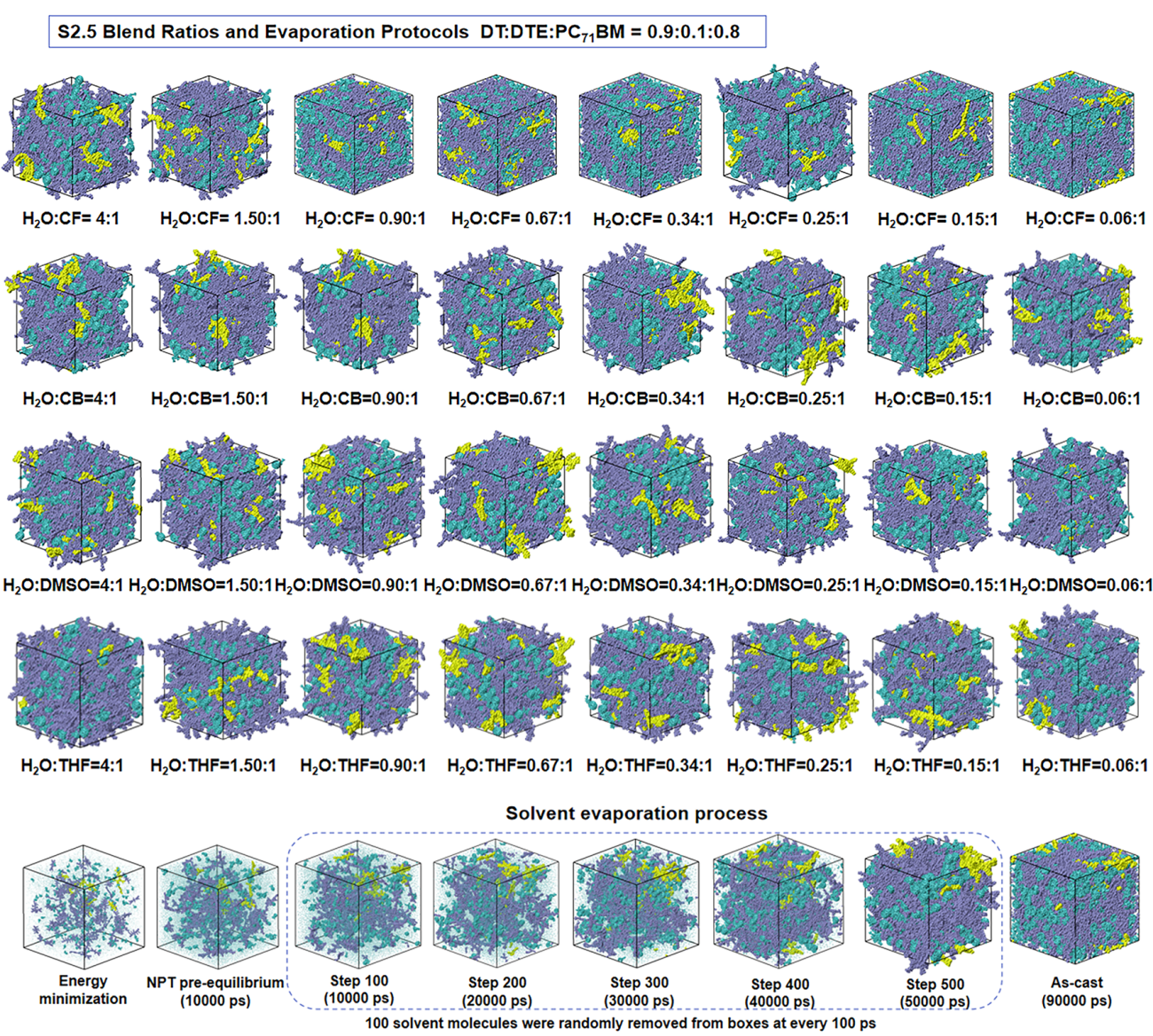


**Figure S5**. Representative and final snapshots of the blending morphologies for DT:DTE:PC_71_BM in different solvent radio of H_2_O and solvents (including CF, CB, DMSO and THF).

# Section S3 Dynamic Alloy-like Morphological Evolution in H_2_O

## Section S3.1 Identification of Critical Process

In order to identify the critical process from cascade model to alloy-like model in H_2_O, the formation process of alloy-like model in MD simulation was searched step by step, as shown in **Figure S6**. That is, trace the simulation process in which “COM *r* of DT-DTE is between DT-PC_71_BM and DTE-PC_71_BM” relationship occurs. The working criterion is a switch in the COM distance order from the cascade type, *r*_DT-PC71BM_ < *r*_DTE-PC71BM_ < *r*_DT-DTE_, to the alloy-like order, *r*_DT-PC71BM_ < *r*_DT-DTE_ < *r*_DTE-PC71BM_. COM *r* order in RDF as a characteristic was used for identifying DTE distribution in **Figure 2c**, which was calculated for each step and time interval (100 ps) during solvent evaporation process.


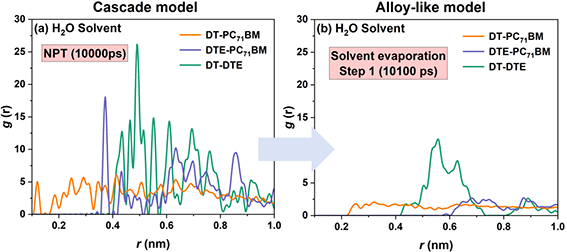


**Figure S6**. Center-of-mass (COM) radial distribution functions (RDFs) between DT, DTE and PC_71_BM identify the critical process from the cascade model to the alloy-like model during MD simulations with H_2_O solvent.

## Section S3.2 Migration Distance and Stacking Molecules Statistics of DTE

COM RDFs were computed at 100 ps intervals during solvent evaporation to locate the first occurrence of this order reversal, which marks the onset of the alloy-like morphology (Figure S5). This quantitative tagging step aligns with the morphological outcomes in Figure 2 and provides the time points used for the MSD and cluster analyses described in this Section.

The simulation time for critical processes is 100 ps during the solvent evaporation process, which is generated by the movement process of DTE molecules. In order to further analyze the reason of DTE entering into the DT phase, the migration distance of DTE is firstly measured by the mean square displacement (MSD) to evaluate the migration distance of 13 DTE molecules in **Figure S7a**. While the migrated distance only illustrates the migration distance but not the migration direction, the molecular clusters were obtained by limiting the distance around the DTE based on the RDF peaks. Therefore, the DT and PC_71_BM contents around the DTE molecules were counted to identify the migration direction of DTE molecules. In **Figure S7b-c**, red and yellow colors denote the initial structure of the critical process, and blue and green colors indicate the final structure of the critical process. The molecular cluster with the most significant change in the number of DT and PC_71_BM and migration distance was used as a screening criterion to select a typical molecular cluster. Therefore, 4^th^ DTE (DTE-4) was selected in H_2_O solvent radio.

**Molecular clusters were obtained as follows:**

1. Counting the MSDs of the thirteen DTE molecules in the MD-simulated blended clusters of ternary systems during the critical process of 0-100 ps ( **Figure S7a**);
2. Limiting the distance around the DTE by the COM distances of the first nearest-neighbour peaks in the RDF as radiuses. 26 DTE-centred molecular clusters were obtained at 0 ps and 100 ps, respectively;
3. Counting the number of DT and PC_71_BM molecules surrounding the DTE in each molecular cluster at 0 ps and 100 ps (**Figures S7b-c**);
4. Screening molecular clusters with high MSD values and fluctuations in the number of DT and PC_71_BM molecules as typical research models (**Figure 2c**).


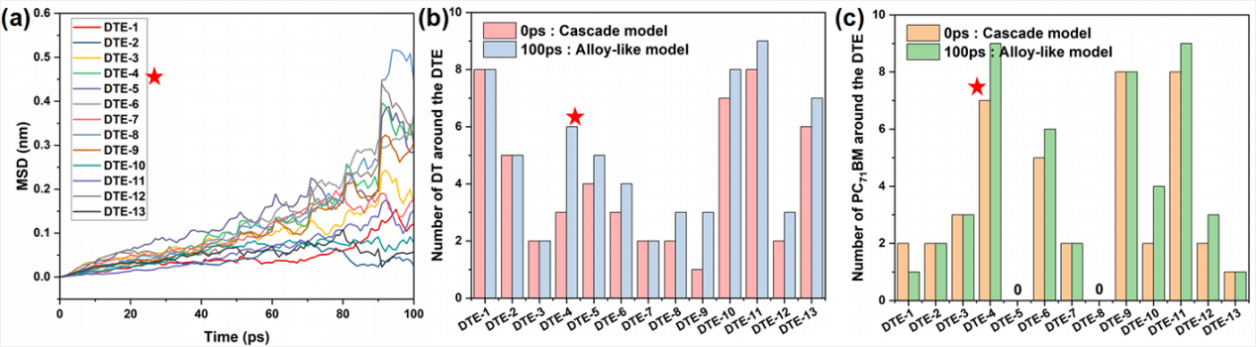


**Figure S7**. Calculated mean square displacement (MSD) of the thirteen DTE molecules in clusters from 0 to 100 ps. The number of (b) DT and (c) PC_71_BM counted around the DTEs at 0 ps and 100 ps. Asterisks indicate typical DTE molecules screened.

## Section S3.3 Hydrogen-Bonding Network

### S3.3.1 Hydrogen Bond Strength Classification

To characterize the evolution of hydrogen-bonding networks (HBNs) during the morphological transition, we categorized all hydrogen bonds based on their estimated bond energies. This classification was designed to ensure both physical relevance and statistical robustness across the simulated trajectory.

The hydrogen bond energy (*E*_HB_) was calculated by Gaussian 16^[11]^ using the electron density at the bond critical point (*ρ*_BCP_) according to the formula proposed by Emamian et al. based on Atoms-In-Molecules (AIM) theory and validated by symmetry-adapted perturbation theory (SAPT) calculations in Multiwfn 3.8 (dev):^[12-15]^

$$\text{E}_{\text{HB}}\text{= −223.08 × }\text{ρ}_{\text{BCP}}\text{ + 0.7423}$$

This formula provides a widely validated and transferable means of estimating H-bond strength from topological electron density analysis. It has been successfully applied to a broad range of weak interactions, including O-H···O, O-H···S, and C-H···π types. This widely adopted relationship has been shown to provide reliable estimations of hydrogen bond strength in diverse systems.^[12]^

### S3.3.2 Statistical Thresholding and Classification

In total, 2527 hydrogen bonds were identified collected over the entire 0-100 ps trajectory, irrespective of donor or acceptor type (i.e., DT, DTE, or PC_71_BM). We extracted the distribution of *E*_HB_ and determined the following percentiles:

25th percentile ≈ -6.58 kcal/mol

Median ≈ -4.81 kcal/mol

75th percentile ≈ -3.12 kcal/mol

Based on this distribution and with reference to empirical criteria summarized by Sobereva^[12]^, we defined the hydrogen bond strength categories as: Strong (*E*_HB_ ≤ -6.5 kcal/mol) means highly directional and persistent hydrogen bonds, Moderate (-6.5 < *E*_HB_ ≤ -3.0 kcal/mol) equals to potentially dynamic and medium strength, and Weak (-3.0 < *E*_HB_ < 0 kcal/mol) denotes transient or surface-level interactions. Interactions with *E*_HB_ ≥ 0 were excluded from hydrogen bond statistics and heatmap visualizations, as they are considered non-attractive under this framework. This classification framework balances physical accuracy (through AIM-derived energy estimation). This classification strategy ensures that our interpretation of HBN dynamics is grounded in physically meaningful energy scales and also reflects the statistical behavior of the current system. The derived classes were directly used in all heatmaps (Figure 3d-f), hydrogen bond proportion plots, and temporal trend analyses in the main text.

# Section S4 Dynamic Alloy-like Morphological Evolution in EtOH

In HBNs, hydrogen bonds are identified by interatomic distances less than the cut-off value of 3.5 Å and hydrogen bond angles less than the angular cut-off value of 40.7°.^[16]^


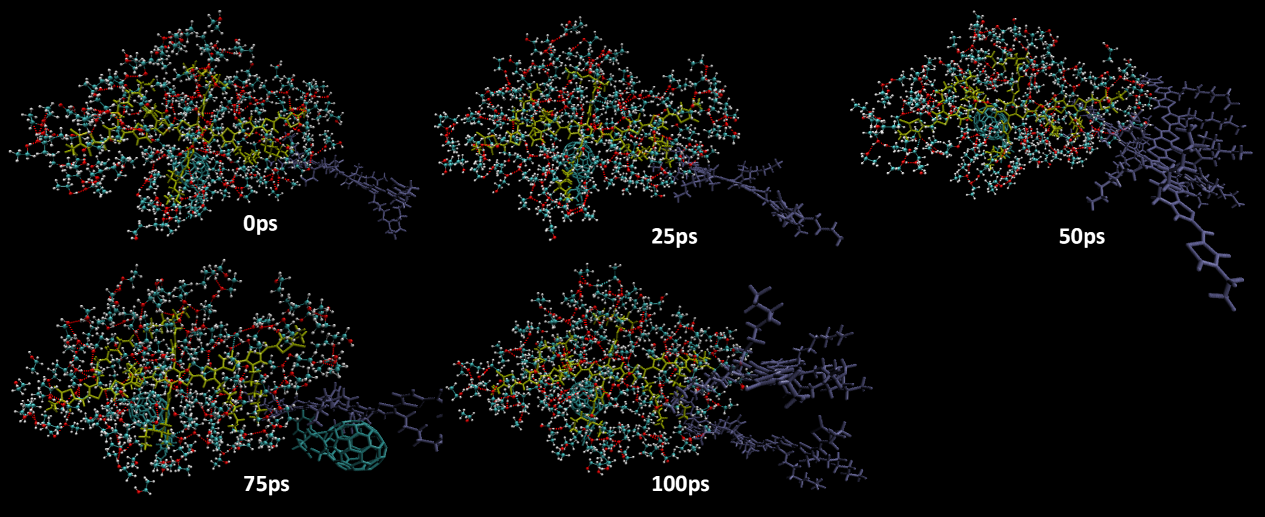


**Figure S8**. Time-resolved morphological evolution and hydrogen-bonding network (HBN) formation during the solvent evaporation process in EtOH environments at 0, 25, 50, 75 and 100 ps, exhibiting fragmented HBNs around DTE clusters.

**Figure 4b** further illustrates the number of hydrogen bonds formed between H_2_O and other molecular components is substantially higher than that of EtOH, supporting a higher interaction density. Moreover, the self-interaction of H_2_O molecules far exceeds that of EtOH (**Figure 4c**), indicating a markedly denser HBN scaffold in aqueous environments. However, the superiority of H_2_O is not merely quantitative. This divergence originates from their molecular hydrogen-bonding topology. H_2_O possesses two hydrogen atoms and two lone pairs, enabling each molecule to act as both donor and acceptor in up to four hydrogen bonds. This facilitates multidirectional, percolative connectivity and supports the construction of extended networks. In contrast, EtOH contains only a single hydroxyl group, restricting its hydrogen-bonding to terminal interactions. Such structural limitations hinder network propagation and result in fragmented solvation patterns incapable of supporting mesoscale morphological reorganization.

# Section S5 Hydrogen-Bonding Network in Non-Fullerene Systems

## Section S5.1 Visualization of Morphological Change Process


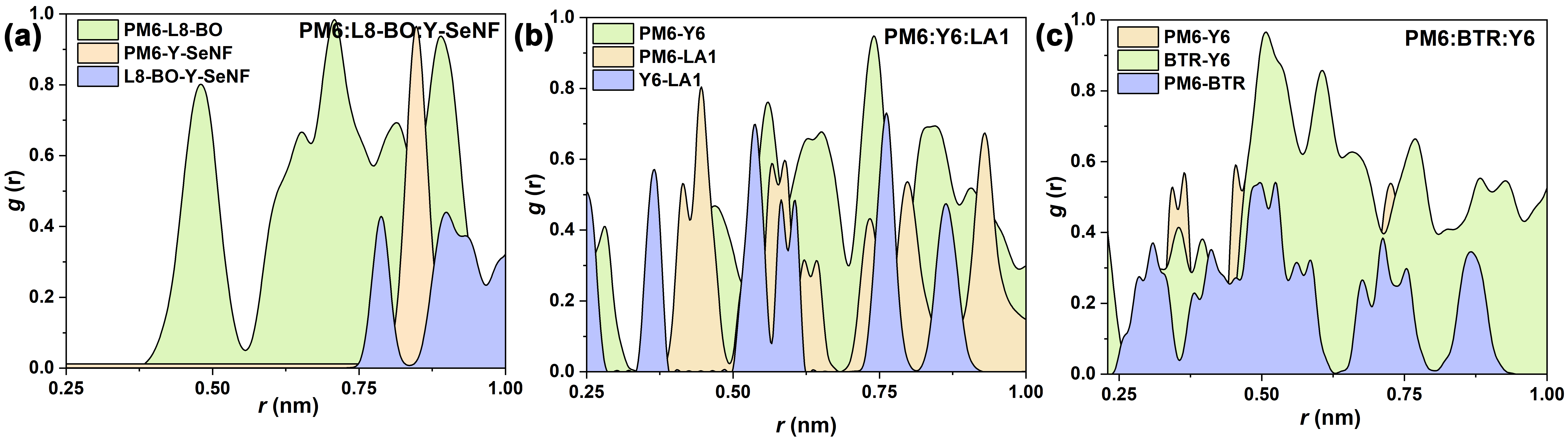


**Figure 9.** Center-of-mass (COM) radial distribution functions for the final equilibral ternary blends of (a) **PM6:L8-BO:Y-SeNF**, (b) **PM6:Y6:LA1**, and (c) **PM6:BTR:Y6** system in H_2_O solvent., illustrating differences in molecular packing and third-component distribution.


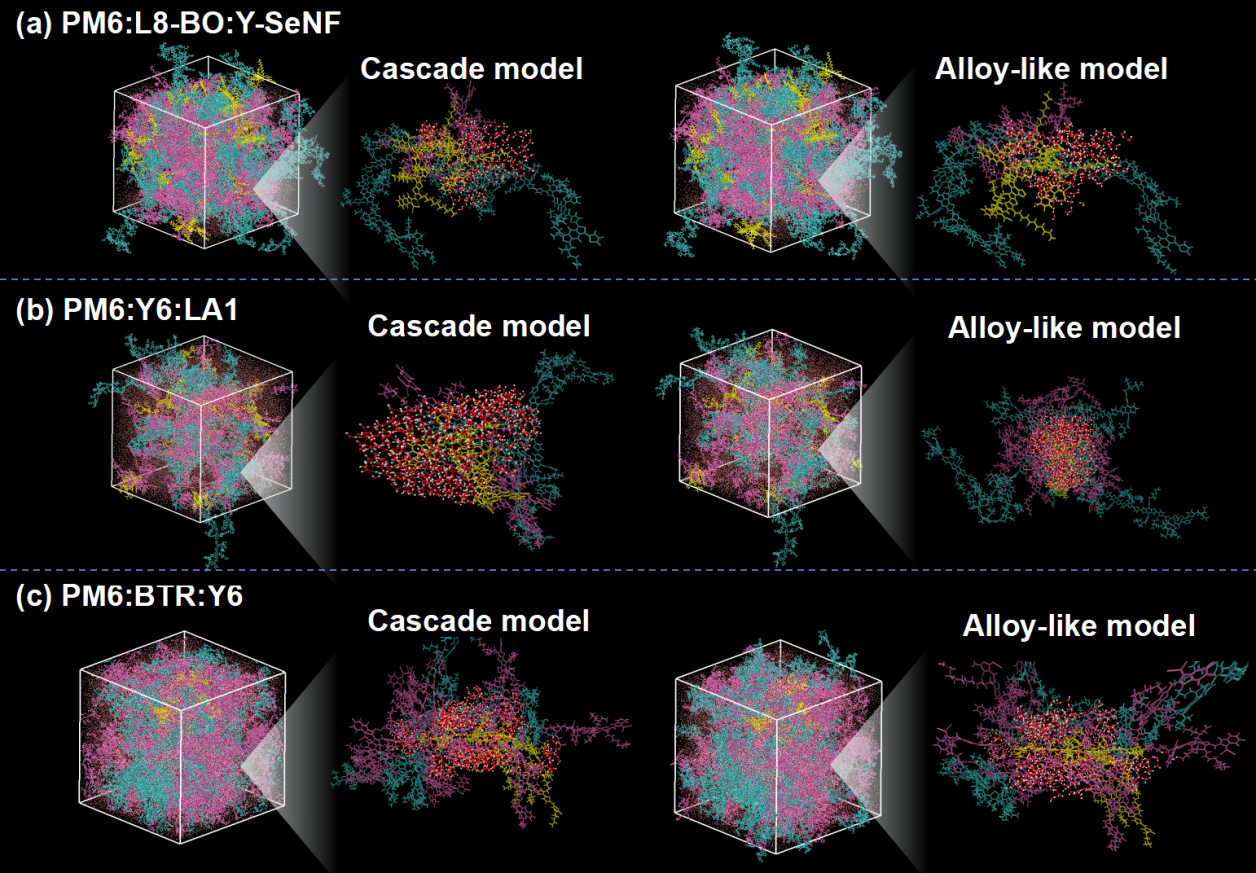


**Figure S10**. Critical process of transition from the cascade model to the alloy-like model with the Hydrogen-bonding network for (a) **PM6:L8-BO:Y-SeNF**, (b) **PM6:Y6:LA1**, and (c) **PM6:BTR:Y6** system in H_2_O solvent. In representative DTE cluster, purple denotes donor PM6, cyan denotes acceptor L8-BO or Y6, yellow denotes the third component Y-SeNF or LA1 or BTR, red denotes H_2_O, and red dashed line denotes hydrogen bonding.

## Section S5.2 Dynamic Elucidation of Morphological Change Process


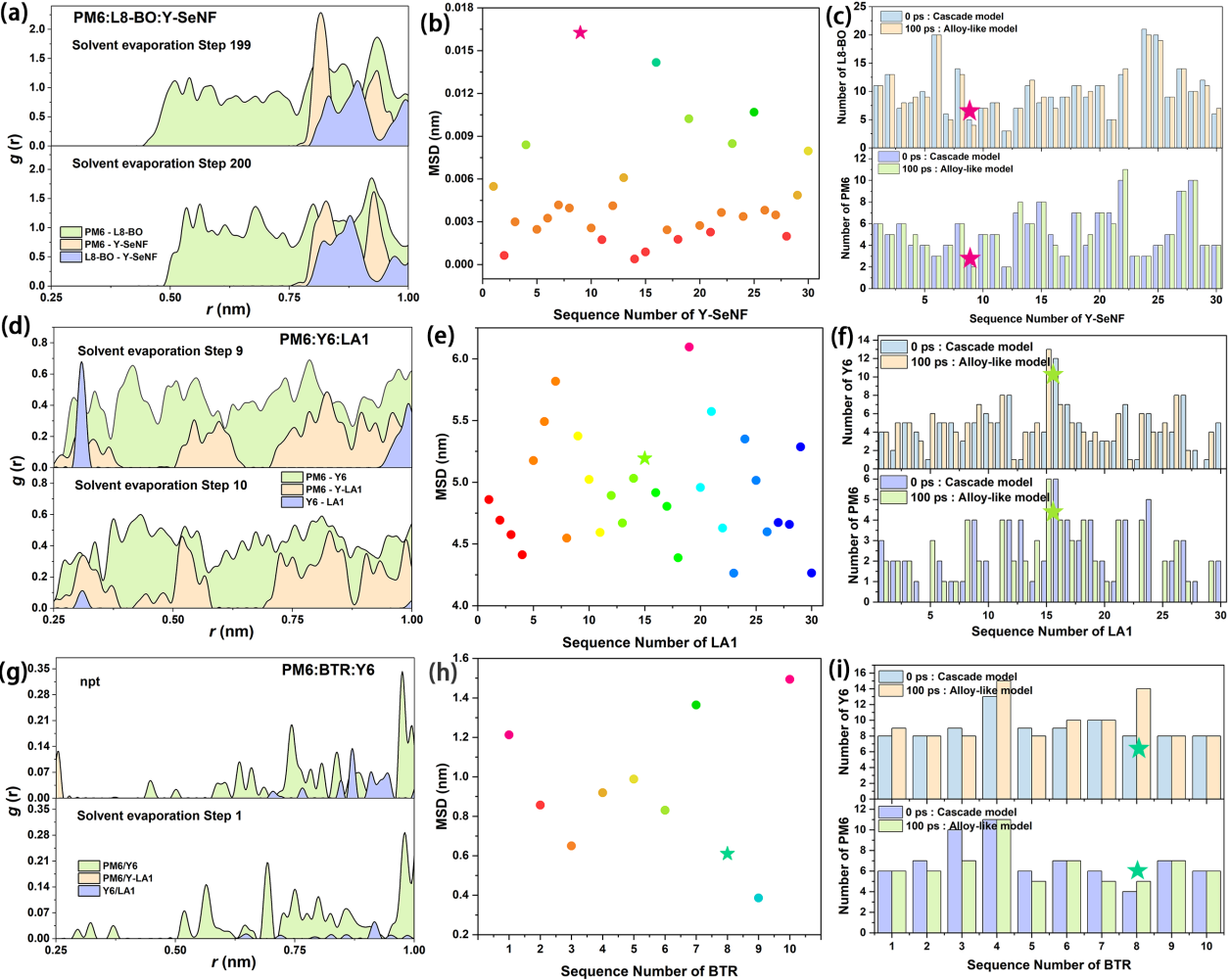


**Figure S11.** Time-resolved snapshots of (a) **PM6:L8-BO:Y-SeNF**, (d) **PM6:Y6:LA1**, and (g) **PM6:BTR:Y6** system under H_2_O solvent evaporation, highlighting the morphological transition from cascade to alloy-like configurations. Mean square displacement (MSD) of representative guest molecules, including (b) Y-SeNF (9^th^ Y-SeNF), (e) LA1 (16^th^ LA1), and (h) BTR (8^th^ BTR), demonstrating directional migration from PM6 toward acceptor (L8-BO or Y6) domains. Bar plot of the number of PM6 and L8-BO, or Y6 acceptor molecules in proximity to (c) Y-SeNF, (f) LA1, and (i) BTR at 0 ps and 100 ps, revealing compositional reorganization consistent with phase embedding.

To address the final part of your comment , we analyzed the critical 200 ps window during which the structural transition occurs, corresponding to step 199 to step 200 in Figure S11, and examined the hydrogen-bonding statistics, solvent accessible surface areas, and representative configurations. The results of this analysis are summarized in Figure S12 of Section S5.2 in the Supporting Information, where hydrogen bonding statistics, solvent accessible surface areas and representative configurations collectively clarify the distinct roles of PM6, Y-SeNF and L8-BO in shaping H_2_O mediated interactions during this transformation.

**Figure S12a** shows the evolution of hydrogen bonds formed between each component and H_2_O during 0-200 ps. L8-BO consistently forms the largest number of hydrogen bonds, followed by Y-SeNF, while H_2_O···PM6 hydrogen bonds remain the fewest throughout the entire evaporation process. The ordering L8-BO > Y-SeNF > PM6 is stable with no crossings or reversals, indicating that the preferential association of H_2_O with the NFAs is not a transient fluctuation but a persistent interaction pattern. The small number of hydrogen bonds involving PM6 reflects its weak solvent accessibility, whereas both Y-SeNF and L8-BO form substantially more hydrogen bonds with H_2_O. In particular, L8-BO exhibits the largest absolute number due to its role of main acceptor, and its population is much larger than that of Y-SeNF as the third component. As a result, both acceptors, rather than PM6, participate in a stronger and more continuous HBN, which provides a basis for the subsequent redistribution of Y-SeNF toward acceptor rich regions. **Figure S12b** presents the total, hydrophobic and hydrophilic solvent accessible surface areas (SASA) of PM6, Y-SeNF and L8-BO. The total SASA decreases in the order PM6 > L8-BO > Y-SeNF, which reflects their molecular sizes. The hydrophilic SASA characterizes the exposure of polar groups to the solvent, follows the distinct order PM6 < Y-SeNF < L8-BO. This confirms that the polar ester groups of PM6 are effectively shielded by its branched alkyl chains, limiting H_2_O access. In contrast, the terminal carbonyl rich regions of Y-SeNF and L8-BO remain more exposed to the mixed solvent. The hydrophilic SASA of L8-BO is the largest, indicating that its polar groups are the most accessible to H_2_O. Y-SeNF shows intermediate accessibility consistent with its hydrogen bond statistics. Above all, the SASA results indicate that water interacts much more strongly with the two acceptors than with PM6, and that H_2_O mediated effects involving Y-SeNF and L8-BO are more pronounced at the molecular interface. **Figure S12c** provides structural evidence supporting the hydrogen bonds and SASA analyses. H_2_O forms an extended network surrounding the acceptor backbones, and frequent localization of H_2_O near the selenophene groups of Y-SeNF is visible, where O-H···Se interactions occur in addition to conventional O-H···O hydrogen bonds with carbonyl groups. The enhanced interaction of H_2_O with the Se containing unit arises from the higher polarizability and more accessible lone pairs of Se compared to S, as well as the geometric exposure of the selenophene group and the reinforcing local electrostatic environment created by nearby C=O groups. Although Y-SeNF is only a less component, these features give it a strong per molecule affinity for H_2_O, while L8-BO provides the largest continuous polar surface for accommodating H_2_O molecules. Consequently, the structural snapshot shows strong H_2_O interactions with both NFAs. This interaction pattern stabilizes Y-SeNF close to L8-BO rather than in PM6 rich domains. These analyses collectively clarify why Y-SeNF is guided toward L8-BO rich regions during the morphological transition.


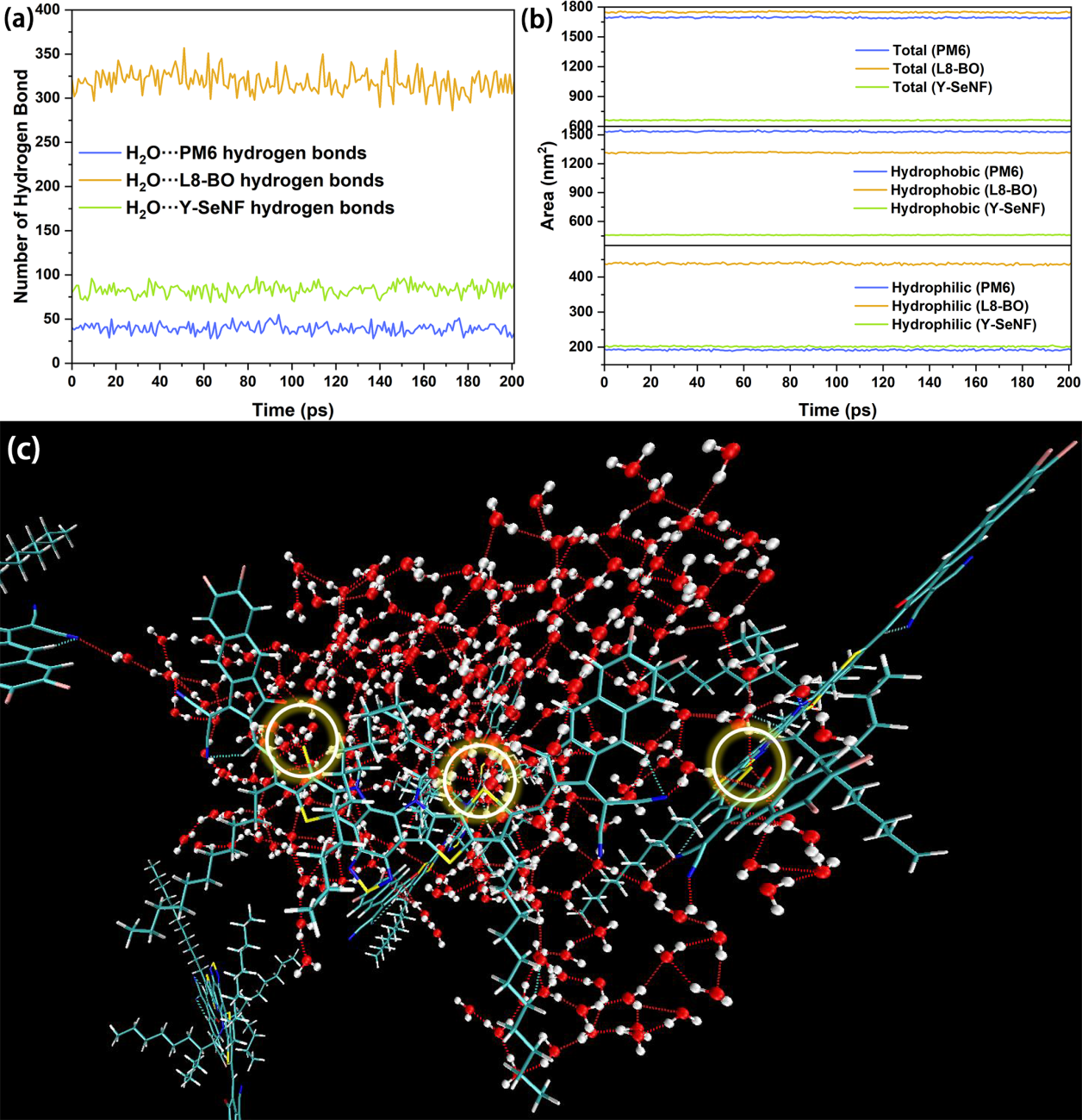


**Figure S12**. (a) Time evolution of hydrogen bond numbers between PM6, L8-BO and Y-SeNF with H_2_O. (b) Solvent accessible surface areas of the three components, including total, hydrophobic and hydrophilic contributions. (c) Representative configuration at the late evaporation stage, showing a continuous hydrogen bond network formed around the Y-SeNF backbones. Yellow circles indicate regions where water molecules accumulate near the selenium atoms of Y-SeNF, demonstrating strong local interactions between water and Se sites.

# Section S6 Co-solvent Strategy

## Section S6.1 Co-solvent Strategy of H_2_O and CF

### Section S6.1.1 Radial Distribution Functions for the Final Equilateral Ternary Blends of mixed H_2_O with CF

As shown in **Figure S13**, the COM *r* orders present differences in the eight groups of solvent ratios, indicating that the DTE distribution is affected by solvent ratios. Among them, the COM *r* order of 0.06:1 ratio is consistent with that in H_2_O solvent, indicating that DTE distribution is dispersed in the DT phase and DT:DTE:PC_71_BM tends to form an alloy-like morphology. The COM *r* orders in the 4:1, 1.50:1, 0.90:1, 0.67:1, 0.34:1, 0.25:1, and 0.15:1 ratios is consistent with the CF solvent, and the COM *r* of DT-DTE decreased with the increase of the ratio of H_2_O solvent. Therefore, the increased proportion of H_2_O solvent could reduce the intermolecular distance between DT and DTE.


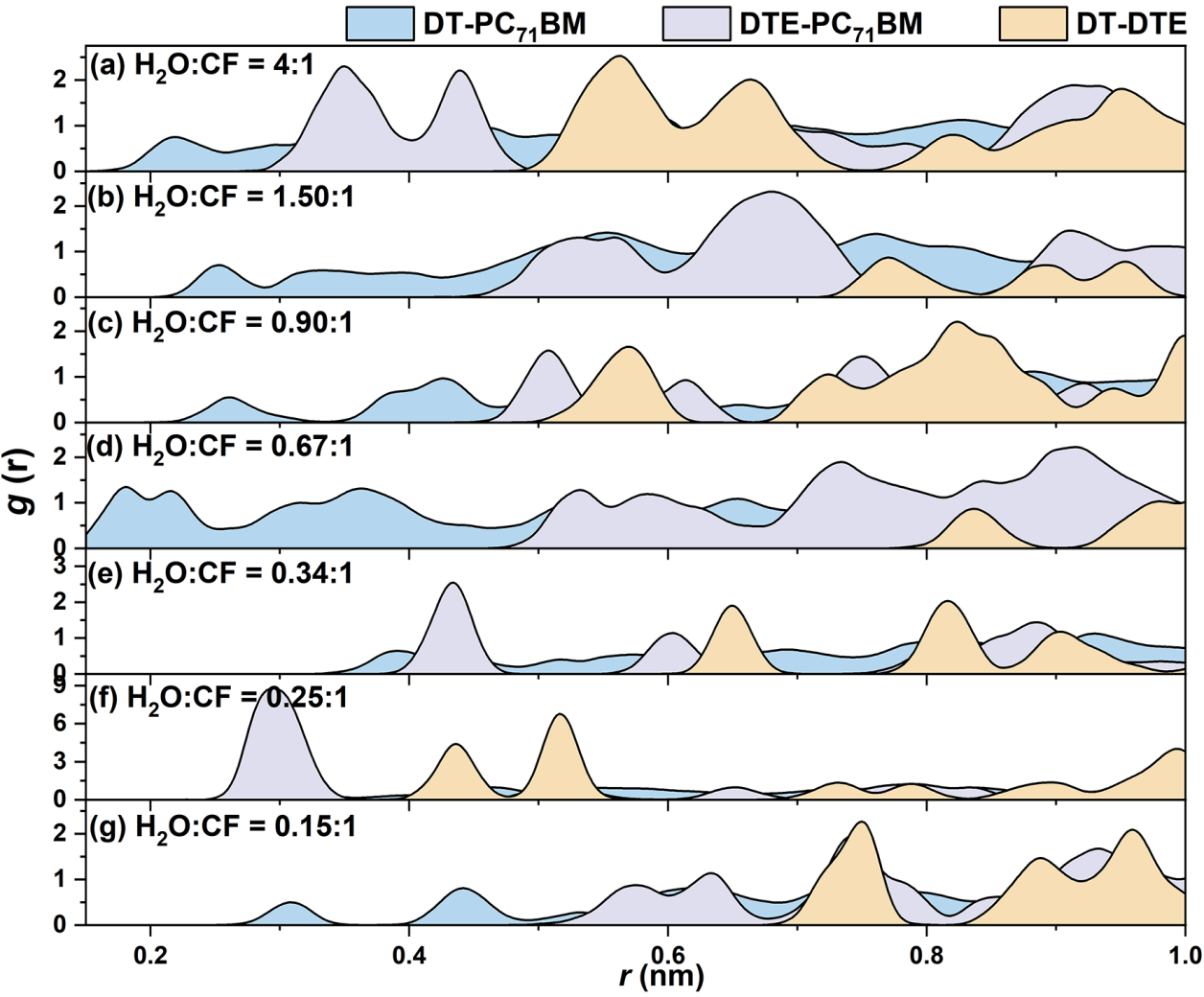


**Figure S13.** COM RDF maps in different solvent radio of H_2_O and CF solvent.

### Section S6.1.2 Dynamic Elucidation of Morphological Change Process


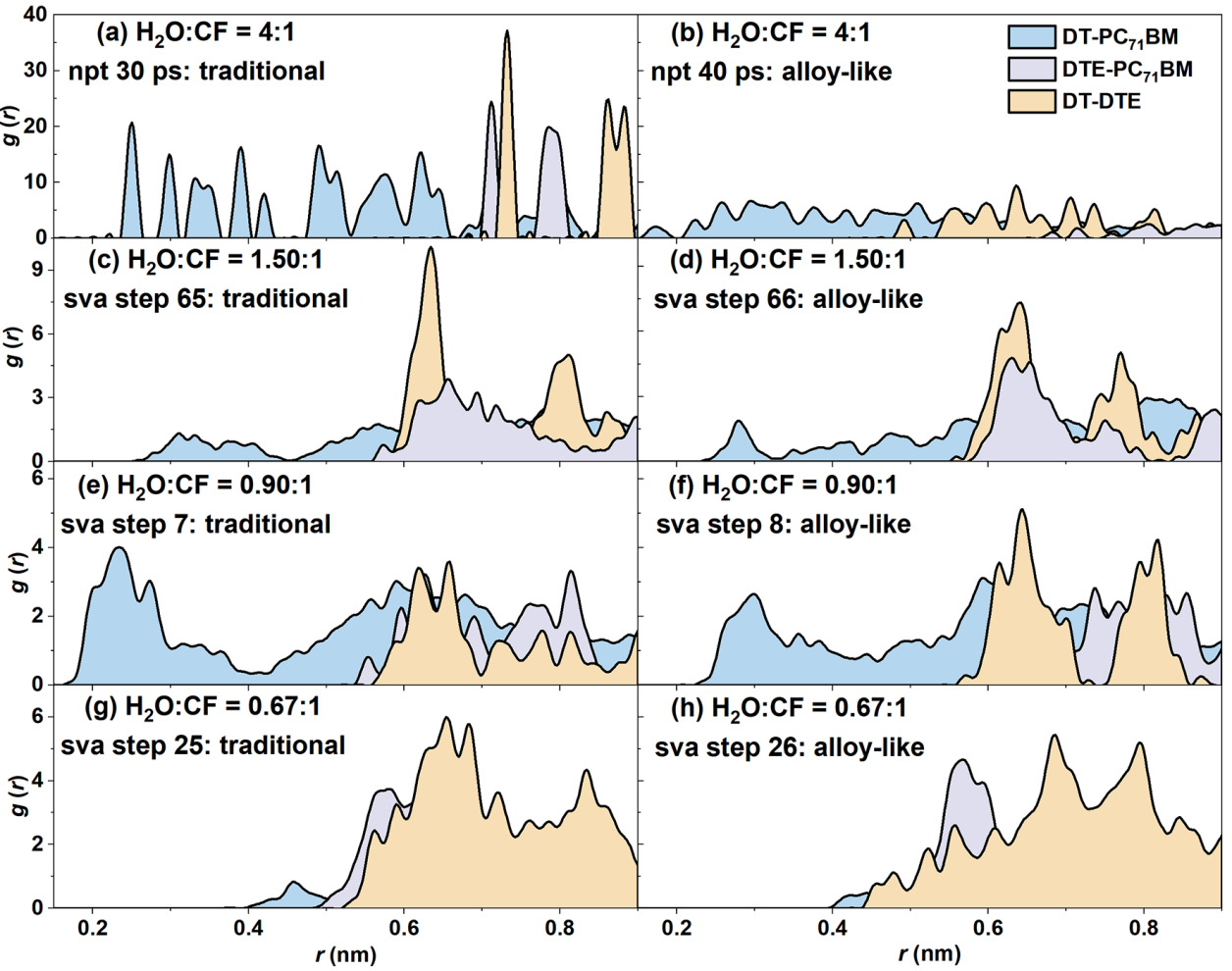


**Figure S14**. Center-of-mass (COM) radial distribution functions (RDFs) between DT, DTE and PC_71_BM identify the critical process from the cascade model to the alloy-like model during MD simulations with 4:1, 1.50:1, 0.90:1, and 0.67:1 solvent ratios


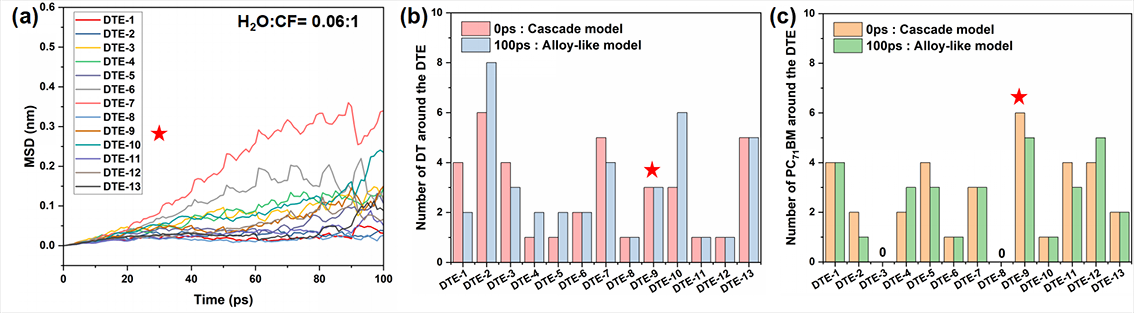


**Figure S15**. (a) Calculated mean square displacement (MSD) of thirteen DTE molecules in clusters from 0 to 100 ps. The number of (b) DT and (c) PC_71_BM counted around the DTEs at 0 ps and 100 ps. Asterisks indicate typical DTE molecules screened.


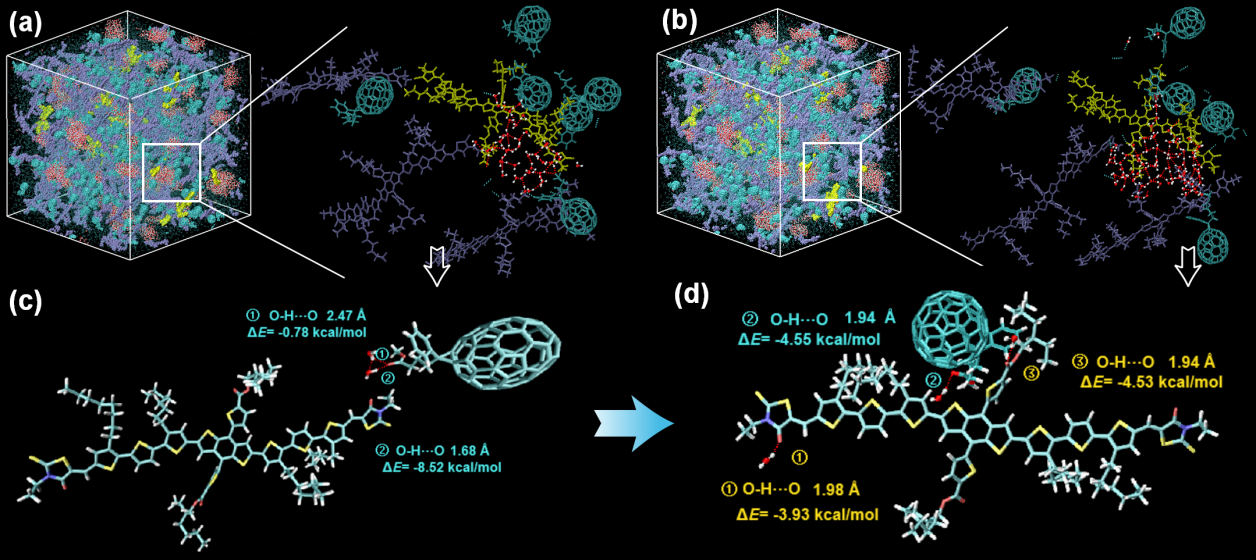


**Figure S16**. Critical process of transition from the (a) cascade model to the (b) alloy-like model with the Hydrogen-bonding network in **0.06:1** solvent radio. Purple denotes DT, yellow denotes DTE, cyan denotes PC_71_BM, blue denotes CF, red denotes H_2_O, and red dashed line denotes hydrogen bonding. In representative DTE cluster, calculated the hydrogen bond energies from (c) cascade model to (d) alloy-like model. Green, white and orange are the hydrogen bonding energies between DT, DTE, PC_71_BM and H_2_O, respectively. -6.5 kcal/mol is strong hydrogen bonds, -6.5~-3.0 kcal/mol are moderately strong, and -3.0~0 kcal/mol are weak hydrogen bonds.

### Section S6.1.3 Hydrogen Bond Counts

Across ratios, similar qualitative hydrogen-bonding patterns are observed, but the overall size and continuity of HBNs decrease as the H_2_O fraction decreases. We quantified the total number of hydrogen bonds formed by H_2_O with each component in representative systems (neat H_2_O and 0.06:1). H_2_O consistently forms more hydrogen bonds with DT due to its larger accessible area, and the H_2_O···DTE hydrogen bonds population increases significantly when network continuity is higher. By contrast, hydrogen bonds involving PC_71_BM remain sparse and transient, reflecting its limited structural integration in the alloy-like morphology.

### Section S6.1.4 Solvent-Accessible Surface Area (SASA)

We evaluated the solvent-accessible surface areas (SASA) of DT, DTE, and PC_71_BM in neat H_2_O, in CF with trace H_2_O, and in the 0.06:1 mixture. SASA was calculated on production trajectories using the Shrake and Rupley algorithm with a 1.4 Å probe, and the sampling and averaging protocols are described in Section S2.5. Figure 5e show that total SASA in CF with trace H_2_O is larger than in neat H_2_O for both the polar component and the nonpolar component. The 0.06:1 system also exhibits higher SASA and aligns with the alloy-like order identified by COM RDFs.

These trends are consistent with the solvation physics. In neat H_2_O the hydrophobic effect favors compact conformations with buried nonpolar cores and reduced exposure. In CF with trace H_2_O hydrophobic segments remain compatible with CF while polar sites recruit the scarce H_2_O to form specific hydrogen bonds, which leads to more extended and more exposed conformations. Across the co-solvent series, higher SASA accompanies shorter DT with DTE COM distance at higher H_2_O fraction and the emergence of a continuous HBN near 0.06:1.

## Section S6.2 The Extension of the Co-solvent Strategy

### Section S6.2.1 Radial Distribution Functions for the Final Equilateral Ternary Blends of mixed H_2_O with CB, DMSO and THF


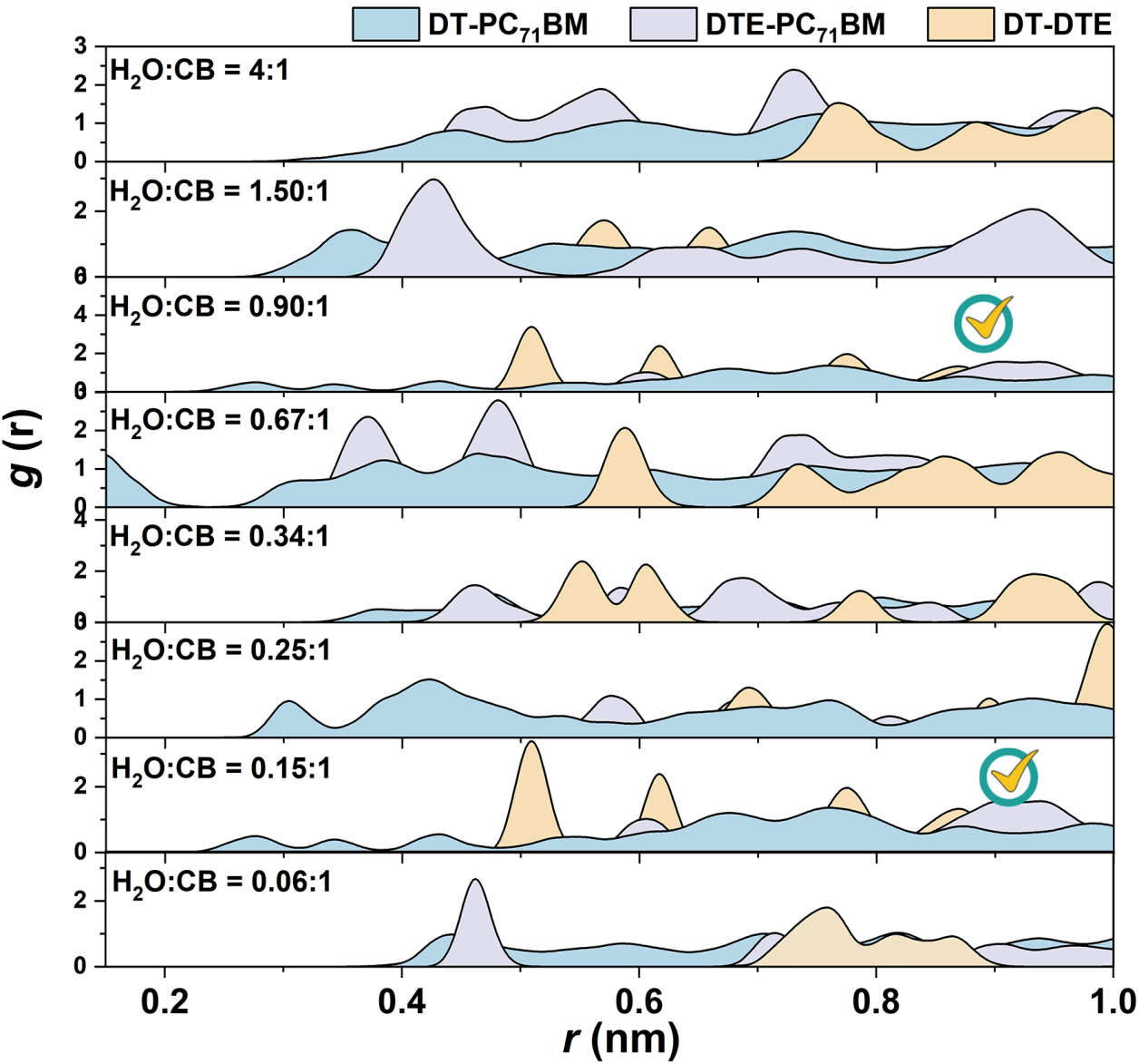


**Figure S17**. COM RDF maps in different solvent radio of H_2_O and CB solvent.


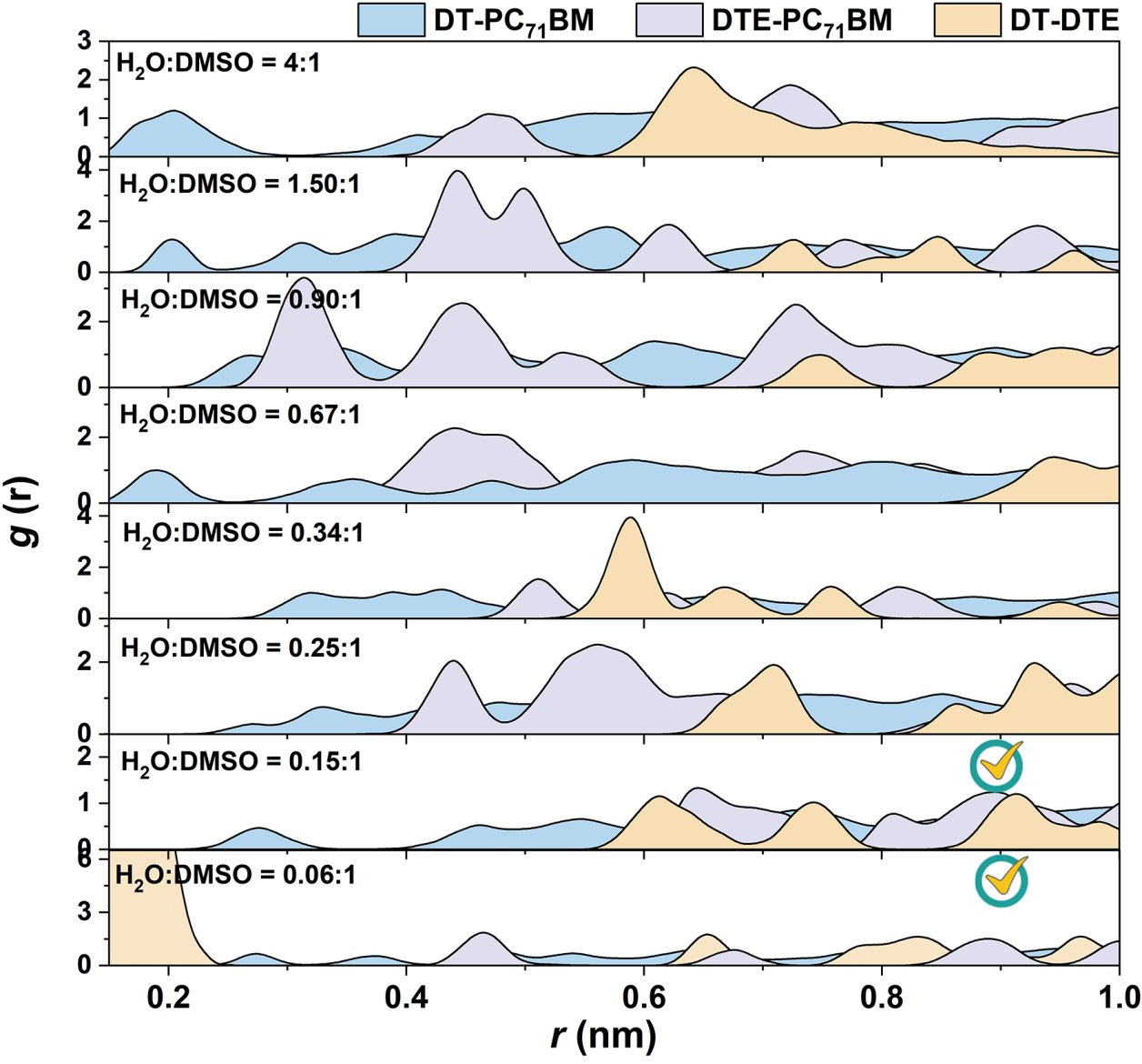


**Figure S18**. COM RDF maps in different solvent radio of H_2_O and DMSO solvent.


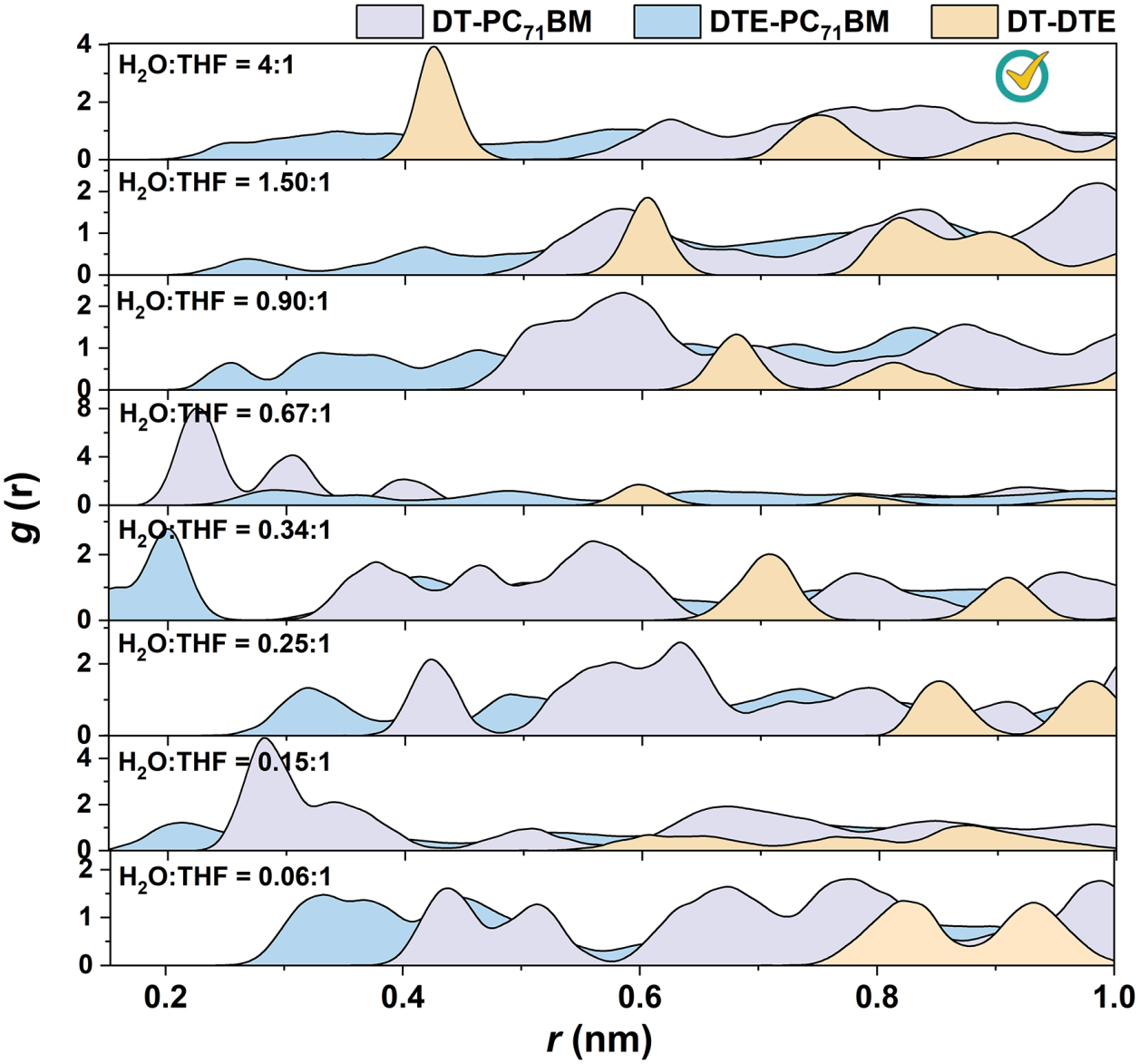


**Figure S19**. COM RDF maps in different solvent radio of H_2_O and THF solvent.

### Section S6.2.2 Dynamic Elucidation of Morphological Change Process of mixed H_2_O with CB, DMSO and THF


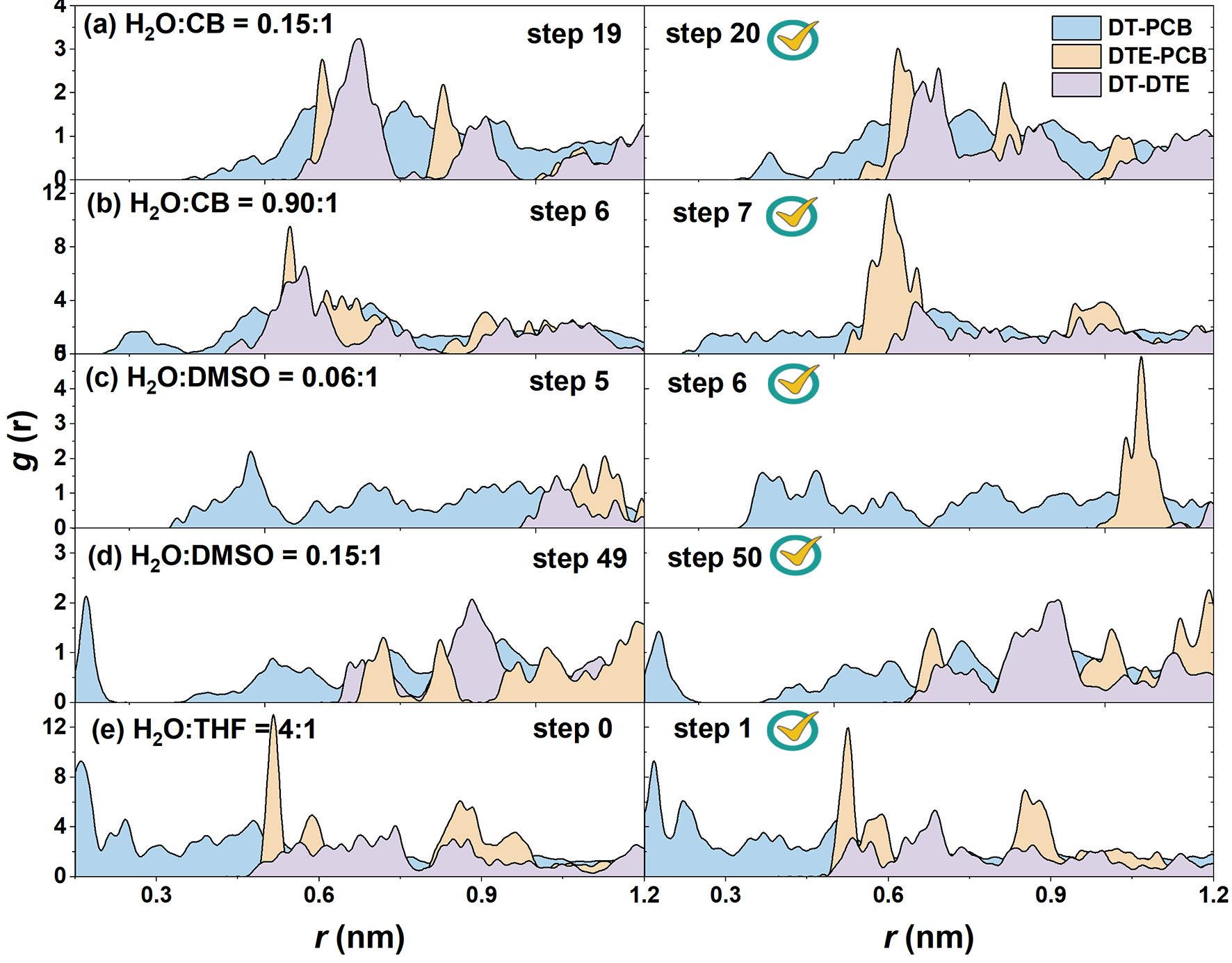


**Figure S20**. Center-of-mass (COM) radial distribution functions (RDFs) between DT, DTE and PC_71_BM identify the critical process from the cascade model to the alloy-like model during MD simulations.


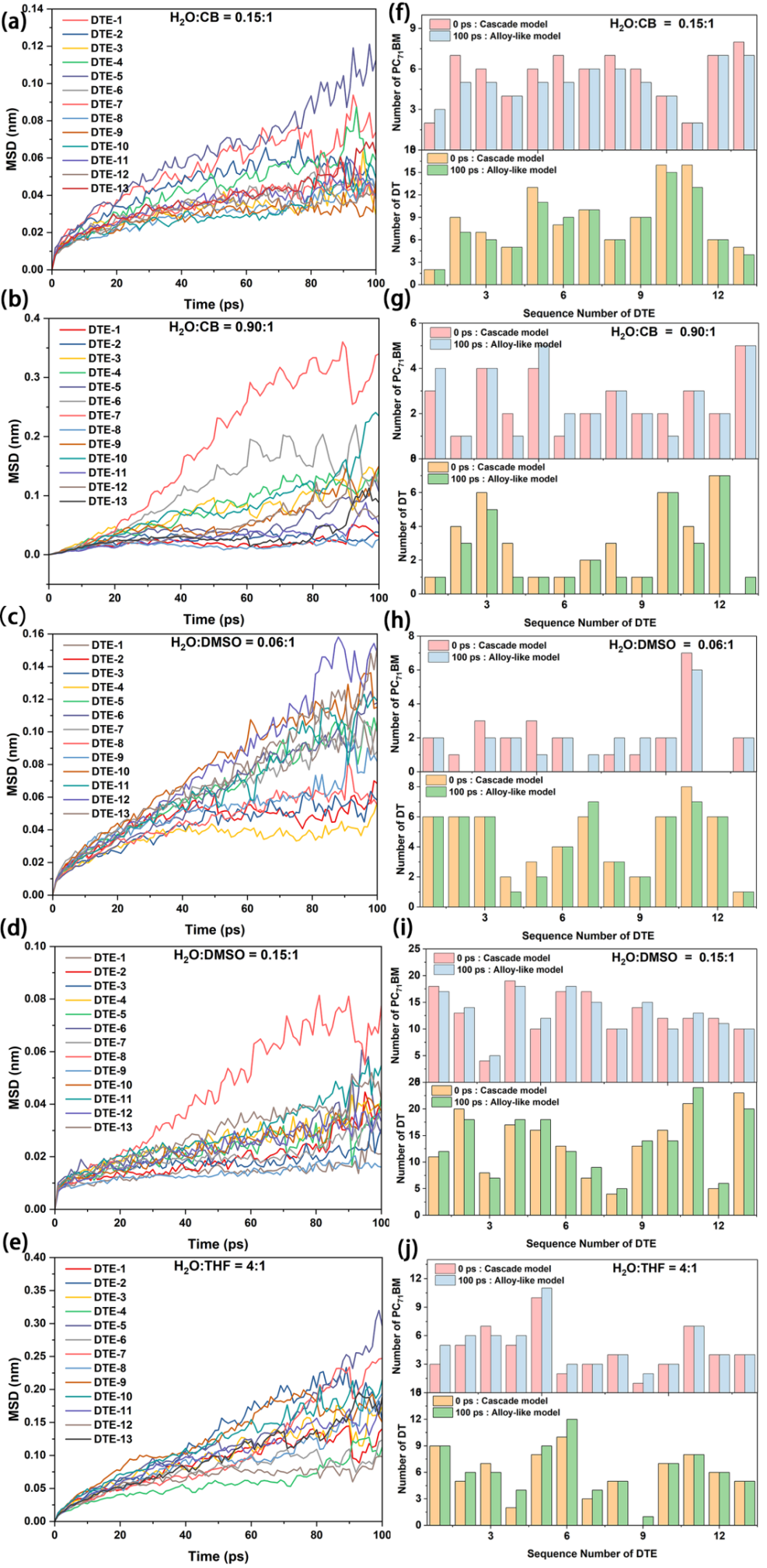


**Figure S21**. (a) Calculated mean square displacement (MSD) of thirteen DTE molecules in clusters from 0 to 100 ps. The number of (b) DT and (c) PC_71_BM counted around the DTEs at 0 ps and 100 ps. Asterisks indicate typical DTE molecules screened.


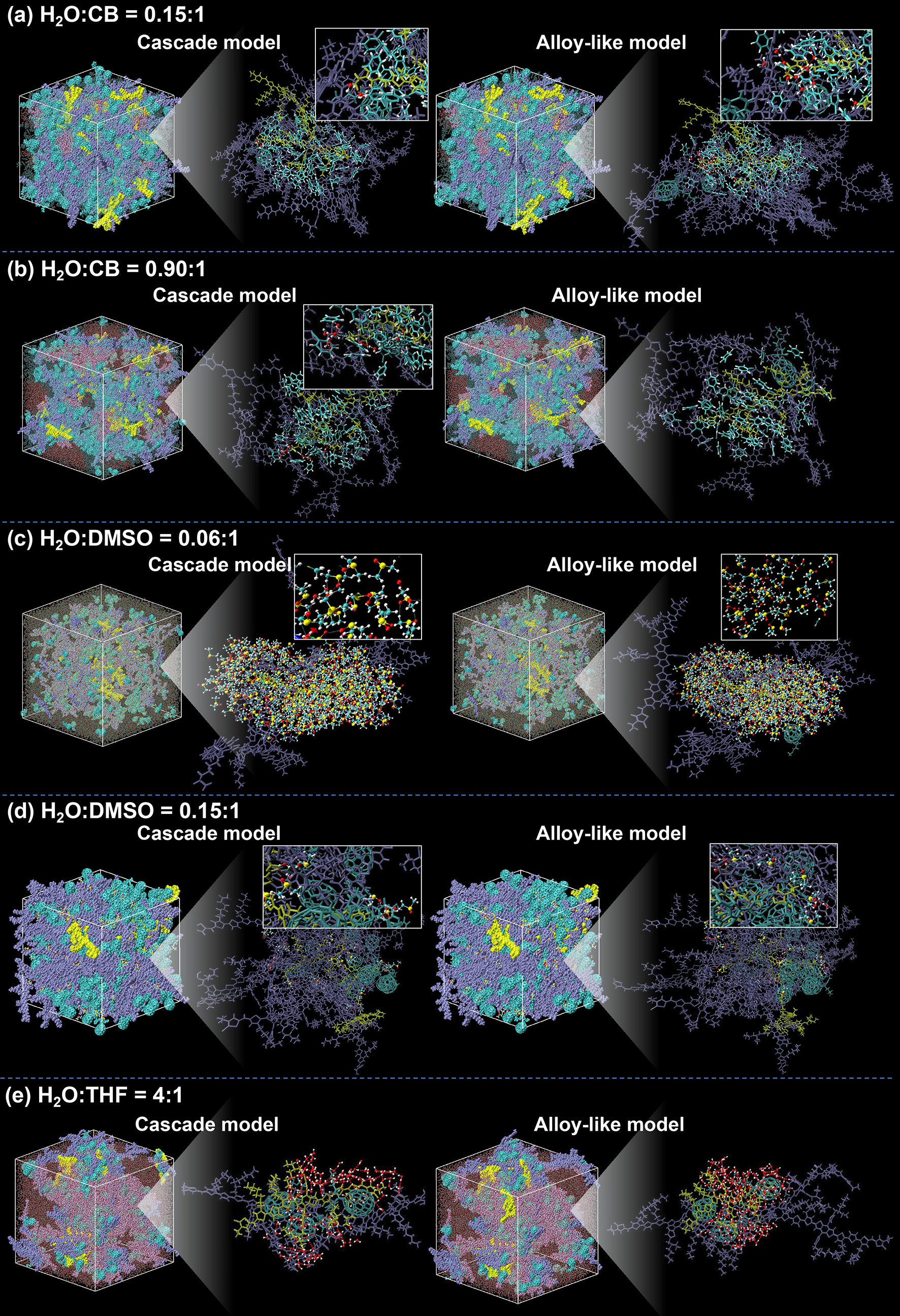


**Figure S22**. Critical process of transition from the cascade model to the alloy-like model with the HBNs in (a) H_2_O:CB = 0.15:1, (b) H_2_O:CB = 0.90:1, (c) H_2_O:DMSO = 0.06:1, (d) H_2_O:DMSO = 0.15:1 and (e) H_2_O:THF = 4:1 radio. Purple denotes DT, yellow denotes DTE, cyan denotes PC_71_BM, blue denotes solvent, red denotes H_2_O, and red dashed line denotes hydrogen bonds.

### Section S6.2.3 Unique Behavior of THF

At low H_2_O content, the strong miscibility between H_2_O and THF disperses the hydrogen bonding motifs and limits H_2_O to solute association. At high H_2_O content, such as the 4:1 system, sufficient water accumulation restores the connectivity required for a continuous HBN.


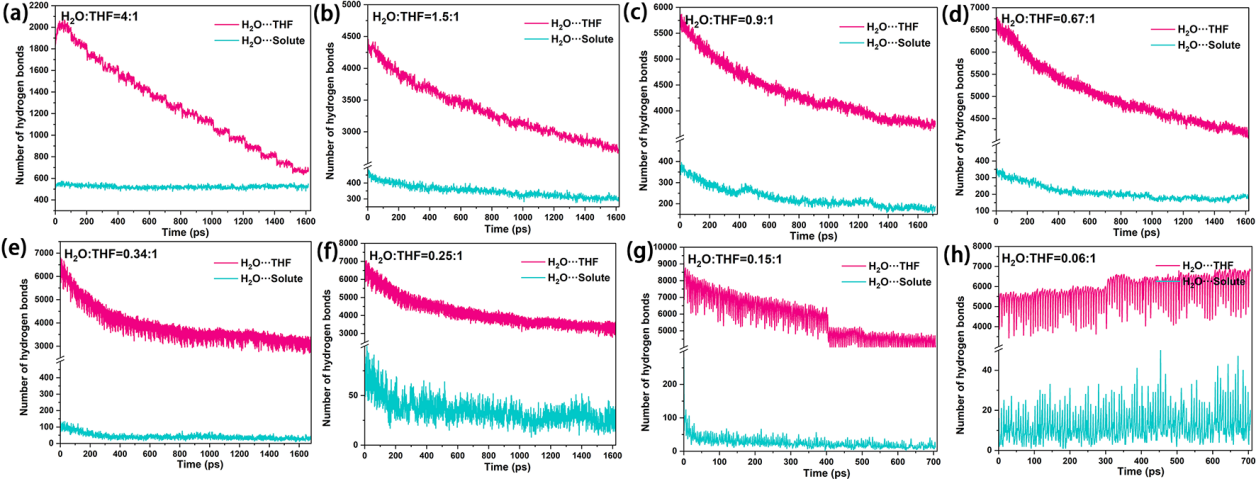


**Figure S23**. Hydrogen bond evolution in mixed H_2_O and THF systems at different volume ratios. Panels a to h correspond to H_2_O:THF ratios of 4:1, 1.5:1, 0.9:1, 0.67:1, 0.34:1, 0.25:1, 0.15:1, and 0.06:1. The pink traces represent the hydrogen bonds formed among H_2_O and THF molecules, and the cyan traces represent the hydrogen bonds formed between H_2_O and THF molecules.

Hydrogen Bonding Features of THF in the H_2_O Rich Environment: The left panel in Figure S23 shows the full simulation box under H_2_O:THF = 4:1 conditions, and the right panels present magnified views of the hydrogen bonding regions. These snapshots display extended water clusters that establish a continuous hydrogen bond network, with THF molecules located around the cluster surfaces while maintaining local hydrogen bonding interactions. At this high H_2_O content, the strong miscibility of THF with water no longer disrupts the network, allowing the hydrogen bonding connectivity to recover sufficiently to support an alloy like configuration in the ternary blend.


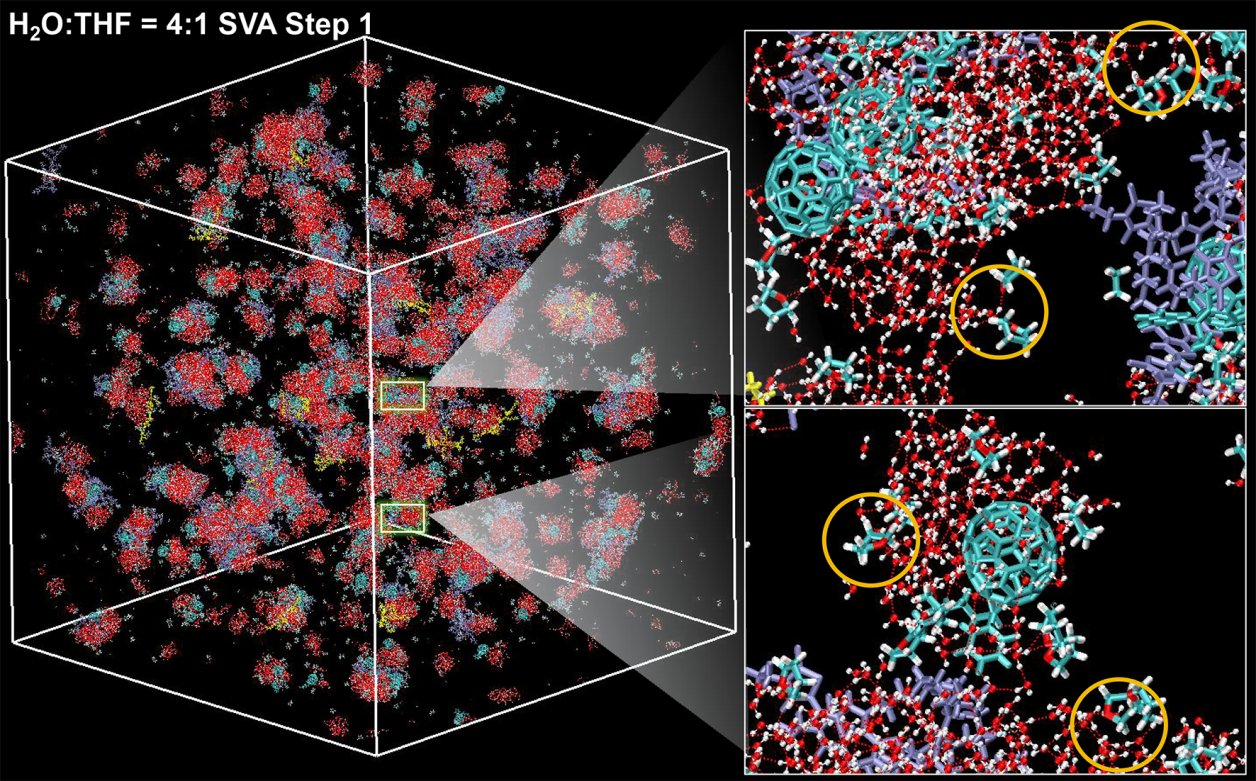


**Figure S24**. Snapshots of the ternary blend under H_2_O:THF = 4:1 conditions. THF molecules gather around water clusters and form local hydrogen bonds.

# Section S7 References

1. Z. Wang, X. Zhu, J. Zhang, K. Lu, J. Fang, Y. Zhang, Z. Wang, L. Zhu, W. Ma, Z. Shuai, Z. Wei, *Journal of the American Chemical Society* **2018**, *140*, 1549-1556.
2. H. Bai, R. Ma, W. Su, T. A. D. Peña, T. Li, L. Tang, J. Yang, B. Hu, Y. Wang, Z. Bi, Y. Su, Q. Wei, Q. Wu, Y. Duan, Y. Li, J. Wu, Z. Ding, X. Liao, Y. Huang, C. Gao, G. Lu, M. Li, W. Zhu, G. Li, Q. Fan, W. Ma, *Nano-Micro Letters* **2023**, *15*, 241.
3. X. Wang, J. Wang, P. Wang, C. Han, F. Bi, J. Wang, N. Zheng, C. Sun, Y. Li, X. Bao, *Advanced Materials* **2023**, *35*, 2305652.
4. X. Kong, L. Zhan, Z. Li, Y. Yang, Y. Liu, H. Qiu, X. Sun, H. Hu, R. Sun, J. Min, S. Yin, W. Fu, H. Chen, *Aggregate* **2024**, *5*, e553.
5. Z. Wang, X. Zhu, J. Zhang, K. Lu, J. Fang, Y. Zhang, Z. Wang, L. Zhu, W. Ma, Z. Shuai, Z. Wei, *Journal of the American Chemical Society* **2018**, *140*, 1549-1556.
6. Z. Wang, G. Han, L. Zhu, Y. Guo, Y. Yi, Z. Shuai, Z. Wei, *Physical Chemistry Chemical Physics* **2018**, *20*, 24570-24576.
7. G. Bussi, D. Donadio, M. Parrinello, *The Journal of chemical physics* **2007**, *126*, 014101.
8. V. Ovchinnikov, S. Conti, E. Y. Lau, F. C. Lightstone, M. Karplus, *Journal of Chemical Theory and Computation* **2020**, *16*, 1866-1881.
9. R. Martonák, A. Laio, M. Parrinello, *Physical review letters* **2003**, *90*, 075503.
10. E. Arunan, G. R. Desiraju, R. A. Klein, J. Sadlej, S. Scheiner, I. Alkorta, D. C. Clary, R. H. Crabtree, J. J. Dannenberg, P. Hobza, H. G. Kjaergaard, A. C. Legon, B. Mennucci, D. J. Nesbitt, *Pure and Applied Chemistry* **2011**, *33*, 25-25.
11. M. J. Frisch, G. W. Trucks, H. B. Schlegel, G. E. Scuseria, M. A. Robb, J. R. Cheeseman, G. Scalmani, V. Barone, G. A. Petersson, H. Nakatsuji, X. Li, M. Caricato, A. V. Marenich, J. Bloino, B. G. Janesko, R. Gomperts, B. Mennucci, H. P. Hratchian, J. V. Ortiz, A. F. Izmaylov, J. L. Sonnenberg, Williams, F. Ding, F. Lipparini, F. Egidi, J. Goings, B. Peng, A. Petrone, T. Henderson, D. Ranasinghe, V. G. Zakrzewski, J. Gao, N. Rega, G. Zheng, W. Liang, M. Hada, M. Ehara, K. Toyota, R. Fukuda, J. Hasegawa, M. Ishida, T. Nakajima, Y. Honda, O. Kitao, H. Nakai, T. Vreven, K. Throssell, J. A. Montgomery Jr., J. E. Peralta, F. Ogliaro, M. J. Bearpark, J. J. Heyd, E. N. Brothers, K. N. Kudin, V. N. Staroverov, T. A. Keith, R. Kobayashi, J. Normand, K. Raghavachari, A. P. Rendell, J. C. Burant, S. S. Iyengar, J. Tomasi, M. Cossi, J. M. Millam, M. Klene, C. Adamo, R. Cammi, J. W. Ochterski, R. L. Martin, K. Morokuma, O. Farkas, J. B. Foresman, D. J. Fox, Wallingford, CT, **2016**.
12. S. Emamian, T. Lu, H. Kruse, H. Emamian, *Journal of Computational Chemistry* **2019**, *40*, 2868-2881.
13. T. Lu, F. Chen, *Journal of Computational Chemistry* **2012**, *33*, 580-592.
14. T. Lu, *Journal of Chemical Physics* **2024**, *161*, 082503.
15. S. Emamian, T. Lu, H. Kruse, H. Emamian, *Journal of Computational Chemistry* **2019**, *40*, 2868-2881.
16. W. Humphrey, A. Dalke, K. Schulten, *Journal of Molecular Graphics* **1996**, *14*, 33-38.

# Appendix: List of Abbreviations and Their Full Names or Definitions

**Table A1.** Full names of all abbreviations used in the manuscript

| **Full name** | **Abbreviation** |
| --- | --- |
| organic solar cells | OSCs |
| power conversion efficiency | PCE |
| water | H_2_O |
| hydrogen-bonding networks | HBNs |
| molecular dynamics | MD |
| 4,8 bis(5 alkylrhodanine 3 yl)benzo[1,2 b:4,5 b']dithiophene with three thienyl linkers | DR3TBDTT |
| 4,8 bis(5 ethylrhodanine 3 yl)benzo[1,2 b:4,5 b']dithiophene with three thienyl linkers | DR3TBDTT-E |
| 6,6-phenyl-C71-butyric acid methyl ester | PC_71_BM |
| DR3TBDTT | DT |
| DR3TBDTT-E | DTE |
| center-of-mass | COM |
| radial distribution function | RDF |
| dimethyl sulfoxide | DMSO |
| chloroform | CF |
| tetrahydrofuran | THF |
| chlorobenzene | CB |
| center-of-mass (COM) distance of the first nearest peak of radial distribution function (RDF) between the DT and PC_71_BM | *r*_DT-PC71BM_ |
| center-of-mass (COM) distance of the first nearest peak of radial distribution function (RDF) between the DTE and PC_71_BM | *r*_DTE-PC71BM_ |
| center-of-mass (COM) distance of the first nearest peak of radial distribution function (RDF) between the DT and DTE | *r*_DT-DTE_ |
| mean square displacement | MSD |
| benzo[1,2-b:4,5-b′]dithiophene | BDT |
| atoms in molecules | AIM |
| ethanol | EtOH |
| also known as PBDB-T-2F, poly[(2,6-(4,8-bis(5-(2-ethylhexyl)thiophen-2-yl)benzo[1,2-b:4,5-b′]dithiophene))-alt-(5,5-(1′,3′-di-2-thienyl-5′,7′-bis(2-ethylhexyl)benzo[1′,2′-c:4′,5′-c′]dithiophene-4,8-dione))] | PM6 |
| 2,2'-((2Z,2'Z)-((12,13-bis(2-ethylhexyl)-3,9-(2-butyloctyl)-12,13-dihydro-[1,2,5]thiadiazolo[3,4-e]thieno[2",3’':4’,5']thieno[2',3':4,5]pyrrolo[3,2-g]thieno[2',3':4,5]thieno[3,2-b]indole-2,10-diyl)bis(methanylylidene))bis(5,6-difluoro-3-oxo-2,3-dihydro-1H-indene-2,1-diylidene))dimalononitrile | L8-BO |
| 2,2'-((((2,2'-((((4,4,9,9-Tetrakis(4-(2-butyloctyl)phenyl)-4,9-dihydro-s-indaceno[1,2-b:5,6-b']dithiophene-2,7-diyl))bis(selenopheno[3,2-b]thiophene-6,2-diyl))bis(5-(2-butyloctyl)-1H-pyrrole-4,2-diyl))bis(methanylylidene))bis(5,6-difluoro-3-oxo-2,3-dihydro-1H-indene-2,1-diylidene))dimalononitrile | Y-SeNF |
| 2,2'-((2Z,2'Z)-((12,13-bis(2-ethylhexyl)-3,9-diundecyl-12,13-dihydro-[1,2,5]thiadiazolo[3,4-e]thieno[2",3’':4’,5']thieno[2',3':4,5]pyrrolo[3,2-g]thieno[2',3':4,5]thieno[3,2-b]indole-2,10-diyl)bis(methanylylidene))bis(5,6-difluoro-3-oxo-2,3-dihydro-1H-indene-2,1-diylidene))dimalononitrile | Y6 |
| **4,8-bis(5-bromo-2-((3-hexylthiophen-2-yl)methoxy)benzothiadiazole)-2,6-bis(4,7-di(5-hexylthiadiazole)-2,1,3-benzothiadiazole)** | LA1 |
| benzodithio-phene terthiophene rhodamine | BTR |
| solvent-accessible surface area | SASA |
| general amber force field | GAFF |
| restrained electrostatic potential | RESP |
| isobaric-isothermal ensemble | NPT |
| van der Waals | vdW |
| particle-mesh ewald | PME |

**Table A2.** Definitions of all abbreviations used in the manuscript

| **Explanation** | **Abbreviation** |
| --- | --- |
| Parameters describing the interactions or geometric relationships between the DT and PC_71_BM molecules are denoted using the hyphenated notation DT-PC_71_BM. For instance, *r*_DT-PC71BM_ refers to the center-of-mass distance between them. | DT-PC_71_BM |
| “DTE-centered clusters” refer to the local molecular assemblies extracted from the larger blended cluster, where each one is defined with a DTE molecule at its core, encompassing the surrounding molecules within a specific cut-off distance. | DTE-centered clusters |
| Hydrogen bonds between H_2_O and DT molecules, denoted as “**H₂O**···**DT hydrogen bonds”**, were identified based on geometric criteria: a donor-acceptor distance of less than 3.5 Å and a donor-hydrogen-acceptor angle greater than 120°. | H_2_O****···****DT **hydrogen bonds** |
| Hydrogen bonds between H_2_O and DTE molecules, denoted as “**H₂O**···**DTE hydrogen bonds”**, were identified based on geometric criteria: a donor-acceptor distance of less than 3.5 Å and a donor-hydrogen-acceptor angle greater than 120°. | H_2_O****···****DTE **hydrogen bonds** |
| Hydrogen bonds between H_2_O and **PC_71_BM** molecules, denoted as “**H₂O**···**PC_71_BM hydrogen bonds”**, were identified based on geometric criteria: a donor-acceptor distance of less than 3.5 Å and a donor-hydrogen-acceptor angle greater than 120°. | H_2_O****···****PC_71_BM **hydrogen bonds**. |
| Guest solvent H_2_O is introduced into the host solvent CF to construct co-solvent system for film-printing process. | co-solvent strategy |
